# No Evidence of Geographical Structure of Salicinoid Chemotypes within *Populus Tremula*

**DOI:** 10.1371/journal.pone.0107189

**Published:** 2014-10-09

**Authors:** Ken Keefover-Ring, Maria Ahnlund, Ilka Nacif Abreu, Stefan Jansson, Thomas Moritz, Benedicte Riber Albrectsen

**Affiliations:** 1 Department of Plant Physiology, Umeå Plant Science Centre, Umeå University, Umeå, Sweden; 2 Department of Entomology, University of Wisconsin-Madison, Madison, Wisconsin, United States of America; 3 Department of Forest Genetics and Plant Physiology, Swedish University of Agricultural Sciences, Umeå Plant Science Centre, Umeå, Sweden; 4 Department of Plant and Environmental Sciences, University of Copenhagen, Frederiksberg, Denmark; Nanjing Forestry University, China

## Abstract

Salicinoids are well-known defense compounds in salicaceous trees and careful screening at the population level is warranted to fully understand their diversity and function. European aspen, *Populus tremula*, is a foundation species in Eurasia and highly polymorphic in Sweden. We exhaustively surveyed 102 replicated genotypes from the Swedish Aspen collection (SwAsp) for foliar salicinoids using UHPLC-ESI-TOF/MS and identified nine novel compounds, bringing the total to 19 for this species. Salicinoid structure followed a modular architecture of a salicin skeleton with added side groups, alone or in combination. Two main moieties, 2′-cinnamoyl and 2′-acetyl, grouped the SwAsp population into four distinct chemotypes, and the relative allocation of salicinoids was remarkably constant between different environments, implying a highly channeled biosynthesis of these compounds. Slightly more than half of the SwAsp genotypes belonged to the cinnamoyl chemotype. A fraction synthesized the acetyl moiety alone (∼7%) or in combination with cinnamoyl (∼2%), and close to forty percent lacked either of the two characteristic moieties, and thus resemble *P. tremuloides* in their salicinoid profile. The two most abundant chemotypes were evenly distributed throughout Sweden, unlike geographical patterns reported for SwAsp phenology traits, plant defense genes, and herbivore community associations. Here we present the salicinoid characterization of the SwAsp collection as a resource for future studies of aspen chemical ecology, salicinoid biosynthesis, and genetics.

## Introduction

**Figure 1:**
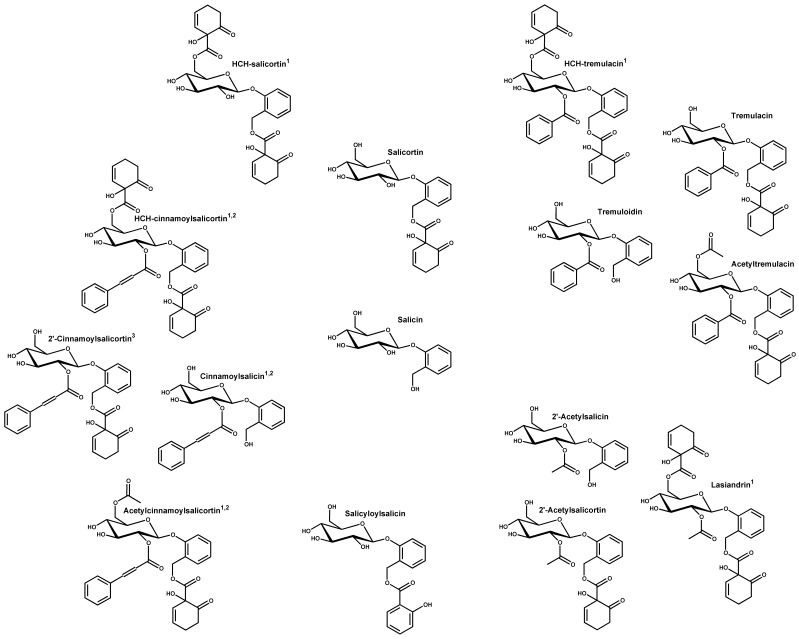
Structural relationship of 19 salicinoids found in the foliage of *Populus tremula* from the SwAsp collection grouped similar to the loading plot (Fig. 2b). 1 =  new compounds for *P. tremula*, 2 =  compounds with two isomers present but the conformation of the cinnamoyl group double bond is ambiguous. 3 =  2′-(*E*)- and 2′-(*Z*)-cinnamoylsalicortin.

Salicinoids, also known as phenolic glycosides [1] or salicylates [2], are dominant bioactive natural products in the Salicaceae (*Populus* and *Salix*) [3]. Salicinoid diversity ranges from simple structures like salicin to higher order compounds, such as cinnamoylsalicortin (Fig. 1). The relationship between herbivores and salicinoids has mostly involved studies of a few dominant compounds that repel generalist insect defoliators [3]–[6] and mammalian browsers [7], [8], attract specialist herbivores [9], [10], or have ambiguous effects on herbivore presence and abundance [11]–[13]. Salicinoids vary in their toxicity, alone and in combination [14], and it has been suggested that they increase in toxicity with greater molecular complexity [6].

Salicinoids derive from the shikimate-phenylpropanoid pathway [3], which produces other phenolic compounds, such as flavonoids, lignins, tannins, and anthocyanins [15], [16]. Several of the genes responsible for specific phenylpropanoid classes have already been described, including those involved in the biosynthesis of monolignols [17], [18], flavonoids [15], [16], and condensed tannins [19], [20]. Salicinoid biosynthesis, however, remains poorly understood [15], [16], [21], [22]. Several evolutionary theories argue that selection should favor a diverse, random, or unpredictable mix of defense compounds in plants [23], [24]. For compounds, such as terpenoids, it has been demonstrated that individual profiles and high diversity at the population level results from only a few key genes, that are expressed consistently within a genotype but vary greatly among genotypes [25], [26]. Although *Populus* and *Salix* species share many salicinoid compounds, their profiles can also separate salicaceous trees both inter- [3], [27] and intraspecifically [28], [29]. Furthermore, studies of hybridizing species suggest that inheritance may often be additive with intermediate levels of phenolic compounds in hybrids [30]–[32], although examples of transgressive inheritance also exist in which hybrids express extreme levels of specific phenolics, including elevated levels of salicinoid compounds [33]. In a recent study, Abreu et al. [29] described an unexpected diversity of salicinoids from five genotypes of European aspen (*Populus tremula* L.). Besides salicortin, tremulacin, salicin, and tremuloidin, the signature salicinoids of the North American sister species *P. tremuloides* [1], [28], *P. tremula* also contained complex salicinoids like 2′-cinnamoylsalicortin, previously only reported for *Salix sericea* [34], and 2′-acetylsalicin and 2′-acetylsalicortin, also found in *S. pentandra* [35]. Abreu et al. [29] showed that concentrations of some of these novel salicinoids in *P. tremula* matched levels in other salicaceous systems that share the same compounds and predicted a minimum of three chemical phenotypes (hereafter chemotypes) of Swedish aspen [29], [36].

Historically, screening work of salicinoids used only qualitative methods, such as thin-layer chromatography [27], [37], [38], as opposed to liquid chromatography-mass spectrometry (LC-MS) techniques presently used for identification and quantification [2], [29], [33], [39]. In addition, species specific profiles have often been based on analyses of a limited number of individuals (e.g., [27], [37], [38]). At the population level, surveying only a few individuals increases the risk of underestimating natural product diversity and abundance. In salicaceous species, hidden salicinoids could thus potentially be a source of reported ambiguous associations found between phytochemistry and biotic stress agents [11]–[13].

With aspen's highly diverse salicinoid assemblage [29], the large genetic diversity in Sweden [40], and its complex associated arthropod communities [41], a careful chemical mapping of a larger population of *P. tremula* is warranted. This study presents an exhaustive identification of salicinoid diversity in 319 individual *Populus tremula* trees replicating 102 genotypes from the Swedish Aspen (SwAsp) collection and evaluates the frequency and distribution of the chemotypes across the landscape. To assess the expression in individual trees, salicinoid profiles of the same genotype were also compared across extreme environments.

## Materials and Methods

### Salicinoid standards

Salicortin, tremuloidin, and HCH-salicortin standards were supplied by Prof. R. L. Lindroth and salicyloylsalicin by Prof. S. D. Mansfield. Salicin and tremulacin standards were purchased from Sigma-Aldrich (St. Louis, MO, USA), and 2′-(*E*)-, 2′-(*Z*)-cinnamoylsalicortin, and tremulacin were isolated from *P. tremula* [39].

### Plant material

Two sets of samples were used for this study, consisting of about ten haphazardly chosen, fully expanded leaves from each of 319 individuals of 102 *P. tremula* genotypes from two different environments, belonging to the Swedish Aspen (SwAsp) collection [29], [36], [41]. SwAsp consists of aspen trees from a range of latitudes (56–67°N) throughout Sweden, collected as roots in 2003 and propagated as genotype replicates. The first greenhouse sample set included leaves from 98 genotypes with 1–2 individuals per genotype that had been growing in standard greenhouse conditions on the Umeå University campus in Umeå, Sweden, since 2005. In 2007, leaves were picked from the greenhouse trees, flash-frozen in liquid nitrogen, and stored at −80°C until chemical analysis (see [29] for details). The second field set, collected in the summer of 2010, consisted of foliage from 41 genotypes from the outdoor SwAsp garden at Sävar (∼20 km NW of Umeå), 37 of which were also represented in the greenhouse population. These trees had been planted in 2004 and at the time of sampling had reached an average height of 220 cm (ranging from 65–405 cm) and every genotype was present in the field in replicates of 2–8. Leaves were harvested and immediately placed in separate glassine envelopes, frozen in liquid nitrogen while in the field, brought to a −80°C freezer, lyophilized in a pre-chilled chamber, and then stored at −20°C until chemical analysis. Replicate individuals of all genotypes currently grow in two common gardens (Ekebo 55.9°N, 13.1°E and Sävar 63.4°N, 20.6°E; see also [36] or [41] for location details of original populations). In addition, most of the genotypes are kept in tissue culture at Umeå Plant Science Centre in Umeå, Sweden, and can be propagated upon request.

### Chemical analysis

Samples were analyzed using ultra high performance liquid chromatography (UHPLC) with UV and electro-spray ionization time-of-flight mass spectrometry (ESI-TOF/MS) detectors, using the same instrumental conditions as Abreu et al. [29]. Sample preparation differed slightly for the two sample sets, using fresh weight material for the greenhouse and lyophilized foliage for the field samples. Frozen greenhouse leaf material was ground in liquid nitrogen using a mortar and pestle. For the 2010 field samples, we ground the lyophilized leaves on a Retsch (Verder Group, Haan, Germany) ball mill, placing crushed foliage into 20 ml plastic vials along with two 12.5 mm carbide balls and shaking them at 60 Hz for 1 minute. For both sample sets, 10.00±1.00 mg powder was extracted in 1 ml of cold (4°C) methanol: chloroform: water (v:v:v), containing deuterated salicylic acid as an internal standard [29]. After centrifugation in a chilled centrifuge, 200 µl of the extract supernatant from greenhouse samples and 100 µl from the field samples was dried in a speedvac. Just before analysis, the dried greenhouse samples were reconstituted with 20 µl of methanol and 20 µl of a 0.1% v/v aqueous formic acid solution and 25 µl of each for the field samples. Differences in the initial amounts of sample dried and final reconstitution volumes served to equalize the final salicinoid concentrations between fresh-frozen and lyophilized leaf tissue, assuming a ∼60 percent difference in water content. Compounds in the reconstituted plant extracts were separated on a C18 UPLC™ column (2.1×100 mm, 1.7 µm) and analyzed by an Acquity photodiode array detector coupled in line with a LCT Premier TOF/MS (all from Waters, Milford, MA, USA) as described in Abreu et al. [29].

MassLynx 4.1 software package (Waters Corp.) allows extraction of single ion chromatograms (±0.15 exact mass unit) from the total ion chromatogram using the QuanLynx module, and was used to search for known and theoretical salicinoids using both the deprotonated ([M-H] ^-^) and formate adduct ([M-H+FA] ^-^) ions (Table S1 in Material S1). QuanLynx software was used to integrate the single ion chromatograms and obtain peak areas, which were normalized with internal standard peak area and individual sample weight. All peak areas represented the formate adduct ion, except for salicyloylsalicin, which included both the deprotonated and formate adduct ion peaks summed, as in Abreu et al. [29].

### Salicinoid identification

Salicortin, tremulacin, salicin, tremuloidin, salicyloylsalicin, HCH-salicortin, 2′-(*E*)-, and 2′-(*Z*)-cinnamoylsalicortin were determined using retention times and molecular weight information of purified standards injected on the UHPLC-ESI-TOF/MS [29], [39]. The compounds 2′-acetylsalicin, 2′-acetylsalicortin, and acetyltremulacin were identified with LC-MS molecular weights and retention times from previous work [29]. The retention times of lasiandrin (HCH-2′-acetylsalicortin) and HCH-tremulacin were confirmed with molecular mass and with *Salix* species known to have these compounds that were included in the present LC runs [35], [42].

UHPLC with high-resolution tandem mass spectrometry (MS/MS) was used to determine the structure of novel salicinoids, using the same chromatographic conditions as Abreu et al. [29]. Peaks were separated on a Hypersil C18 GOLD column (2.1×50 mm, 1.9 µm) using a Thermo Accela LC system coupled to a LTQ Orbitrap MS (all from Thermo Fisher Scientific, Bremen, Germany) and centroid mass spectra of negative ions were collected after collision-induced dissociation (CID) in the LTQ cell at 35 eV. The deprotonated ion was used for all compounds, except for cinnamoylsalicin, which was fragmented using the formate adduct ion.

### Statistical analyses

Salicinoid chemotypes were identified using principal component analysis (PCA; SIMCA-P+ v. 12.0 [43]). Due to differences in sampling environment, we used percentages derived from the normalized peak areas of the 19 salicinoids. To further statistically examine the differences between compound profiles, SAS software version 9.1 [44] was used to perform a two-factor multivariate analysis of variance (MANOVA; PROC GLM function with the MANOVA statement) using the same data as above, with sample set (greenhouse or field grown trees) and chemotype (four chemotypes) as factors. Significant MANOVA tests were followed up with ANOVAs for individual compounds.

Amounts of the most common and abundant salicinoids (salicortin and tremulacin) from the field samples were correlated (PROC CORR) separately for trees either low (TL chemotypes) or high in 2′-cinnamoylsalicortin (CN chemotypes), followed by Fisher's Z to determine if the two correlation coefficients differed.

To compute salicinoid clonal repeatability (*H* ^2^, broad-sense heritability), we used the R statistical package as described by Robinson et al. [44], [45]. Clonal repeatability was calculated the greenhouse and the field samples separately, and for the combined population, when they occurred in replicate of two or more. For all statistical analyses we insured that variables met assumptions of normality, applying transformations where necessary.

## Results

### New salicinoids from *P. tremula*

In addition to the ten salicinoids described from *P. tremula* by Abreu et al. [29], we found nine new compounds after searching the TOF/MS chromatograms of greenhouse and field foliage samples for 55 known and theoretical ions (Fig. 1, Material S1). The new salicinoids included five molecules similar to existing *P. tremula* compounds but with an additional HCH (hydroxycyclohexen-on-oyl) moiety, including HCH-salicortin, HCH-tremulacin, lasiandrin (HCH-2'-acetylsalicortin), and two isomers tentatively identified as HCH-cinnamoylsalicortin. In addition, we found the newly described 2′-(*Z*)- and 2′-(*E*)-cinnamoylsalicortin isomers [29], [39] and two salicinoid isomer pairs tentatively identified as cinnamoylsalicin and acetylcinnamoylsalicortin. Considering the structure of many other salicinoids [3], [39], the additional acetyl and HCH groups are most likely attached to C-6′ of glucose and the new isomer pairs probably contain 2′-(*Z*)- and 2′-(*E*)-cinnamoyl groups, respectively. The UV profiles of the new compounds showed typical salicinoid spectra (Table 1, Fig S1a page 4–8 in Material S1). As with these other studies, all new salicinoids with a cinnamoyl moiety had higher second maxima (274–279 nm) compared to those without this functional group (270–274 nm).

**Table 1:** UV maxima, theoretical and experimental exact masses, molecular formulas, and main high-resolution MS/MS fragments of the new salicinoids from *Populus tremula*.

Compound	λ_max_	*m/z* [M-H]^-^			MS/MS fragments (relative intensity)^a^
		Theoreticalmass	LTQ Orbitrap		
			Mass	Formula	
HCH-salicortin	218, 271	561.1614	561.1611	C_27_H_29_O_13_	423 (100), 477 (58.3), 405 (40.0), 299 (13.9), 437 (9.7), 339 (7.3), 293 (6.2), 231 (5.5)
HCH-tremulacin	221, 272	665.1876	665.1859	C_34_H_33_O_14_	527 (100), 509 (33.6), 543 (28.6), 405 (14.4), 581 (8.4), 403 (2.8), 389 (1.5), 553 (1.0)
Cinnamoylsalicin	218, 279^b^, 219, 279^c^	415.1398	415.1385	C_22_H_23_O_8_	415 (100), 147 (50.9), 309 (39.9), 414 (10.2), 285 (6.6), 509 (6.5), 252 (6.4), 515 (6.4)
Acetylcinnamoylsalicortin	220, 274^b^, 218, 278^c^	595.1821	595.1793	C_31_H_31_O_12_	447 (100), 423 (15.9), 567 (5.2), 213 (4.3), 285 (4.1), 267 (4.0), 471 (3.5), 341 (2.9)
HCH-cinnamoylsalicortin	222, 277^b^ 219, 277^c^	691.2032	691.2023	C_36_H_35_O_14_	553 (100), 535 (28.7), 543 (19.5), 405 (10.2), 484 (6.6), 509 (6.5), 252 (6.4), 515 (6.4)
Lasiandrin	219, 270	603.1719	603.1722	C_29_H_31_O_14_	465 (100), 447 (22.4), 519 (8.5), 561 (5.8), 543 (4.5), 405 (3.6), 341 (3.0), 423 (2.1)

See Fig. S1a in Material S1 for MS/MS spectra.

a =  MS/MS performed on deprotonated isomer 2 of cinnamoyl compounds, except for cinnamoylsalicin, which used the formate adduct [M+FA-H]^-^ (experimental mass *m/z* 461.1433) of isomer 2; b =  isomer 1, c =  isomer 2 by UHPLC retention times.

The MS/MS spectra of the new *P. tremula* salicinoids produced predictable fragments due to similar disassociation mechanisms (Figure S1b page 9–11 in Material S1). The spectra of all compounds with an additional HCH group [HCH-salicortin, and HCH-tremulacin, lasiandrin (HCH-2′-acetylsalicortin), and HCH-cinnamoylsalicortin] had a single dominant fragment due to the loss of the HCH group (neutral loss of 138) from either the core salicyl group or from the glucose. For all compounds, the *m*/*z* of this remaining fragment corresponded to their respective deprotonated salicinoid precursors (salicortin, tremulacin, 2′-acetylsalicortin, and 2′-cinnamoylsalicortin, respectively). The next most common fragment for all HCH-containing salicinoids, except for HCH-salicortin, also involved loss of the HCH group. In this case however, the C-O bond of the ether linkage broke proximal to the HCH moiety. For all compounds, these fragments were *m*/*z* 18 less than breakage of the distal C-O ether bond, indicating dehydration. While this fragmentation mechanism also produced a significant ion for HCH-salicortin (*m*/*z* 405), its second most abundant MS/MS fragment arose from cleavage and loss of most of the cylcohexeneone ring of either of the compound's HCH groups. The other HCH salicinoids also had relatively abundant fragments due to this breakage pattern. In addition, the spectra of all compounds contained a *m*/*z* 405 ion; a secondary fragment resulting from a combination of a loss of an HCH group combined with the cleavage of any moiety present at C-2′ of the sugar. A single ion dominated the MS/MS spectra of acetylcinnamoylsalicortin, resulting from the loss of the cinnamoyl group. Subsequent loss of the acetyl moiety led to the appearance of a deprotonated salicortin ion as the second most abundant ion (*m*/*z* 423). Lastly, fragmentation of cinnamoylsalicin with a formate adduct yielded the deprotonated compound as the primary fragment. Further dissociation of this structure created ions corresponding to a cinnamoyl group and to cinnamoyl-β-D-glucopyranose with subsequent loss of the salicyl moiety.

### Chemotypes and compound relationships

**Figure 2:**
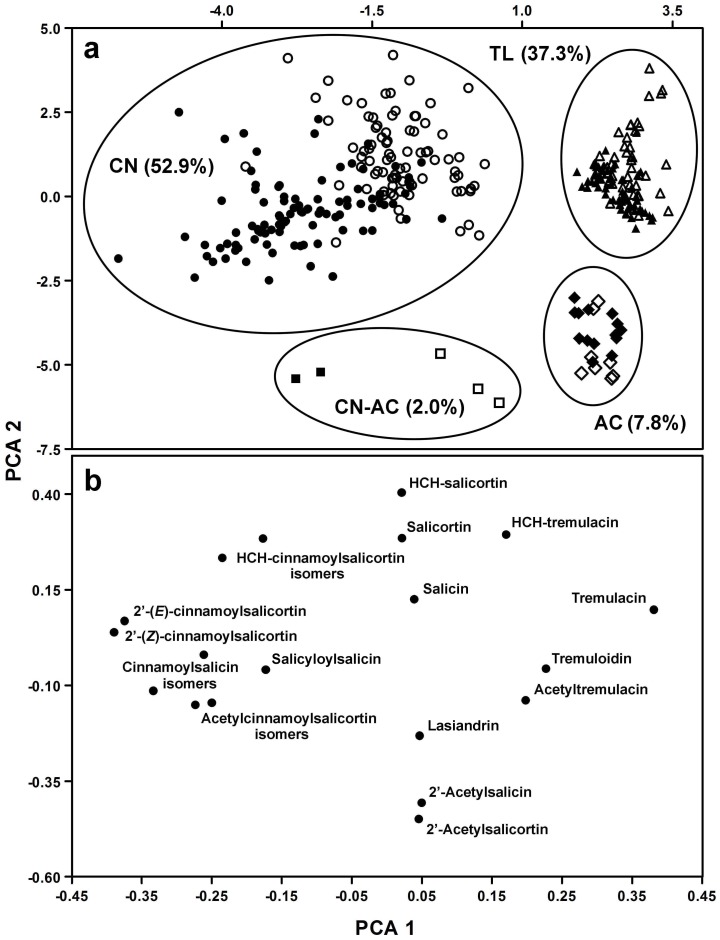
PCA results for chemotypes and compound relationship of 19 salicinoids found in the foliage of *Populus tremula* from the SwAsp collection. a. Score scatter plot of the first two principle components for 319 individuals from 102 genotypes. Solid symbols  =  greenhouse grown trees, open symbols  =  field grown trees; circles  =  CN (2′-cinnamoyl), diamonds  =  AC (2′-acetyl), squares  =  CN-AC (2′-cinnamoyl/2′-acetyl), and triangles  =  TL (*tremuloides*-like) chemotypes. Percentages of clones of the different salicinoid chemotypes in parentheses. **b.** Loading scatter plot of the first two principle components for 19 salicinoids.

All samples contained the signature set of salicinoids in aspen (salicortin, tremulacin, salicin, and tremuloidin) [1], [3], [27], and in agreement with Abreu et al. [29] a subset of the samples also included novel salicinoids. The salicinoid profiles of SwAsp are presented by genotype and environment in Material S2. PCA analysis separated SwAsp trees into four distinct chemotype groups on the basis of 19 salicinoids with 31.2% of the variation explained by PC1 and 18.4% by PC2 (49.6% cumulative; Fig. 2). These chemotypes were mainly defined by the presence of high amounts of salicinoids with either cinnamoyl moieties (CN, 53% of all genotypes), 2′-acetyl moieties (AC, 8%), both of these moieties (CN-AC, 2%), or very low amounts of either (37%; Fig. 2a). The salicinoid profile of the last “*tremuloides*-like” (TL) chemotype resembles that of *P. tremuloides* [46]. Most of the AC chemotype trees had very low levels of cinnamoyl-containing salicinoids, however, five individuals from two genotypes had relatively high levels of compounds with both of these moieties, resulting in the separate chemotype designated CN-AC (Fig. 2a). While trees from particular chemotypes generally grouped together in greenhouse and field grown trees, the PCA showed some divergence between the two environments. This was especially evident for individuals from the CN and CN-AC chemotypes and less so for AC and TL trees (Fig. 2a).

The loading scatter plot of the 19 salicinoids from *P. tremula* showed that most compounds grouped according to specific chemical moieties (Fig. 2b). The first component (PC1) separated compounds with a cinnamoyl side chain [2′-(*E*)- and 2′-(*Z*)-cinnamoylsalicortin and the cinnamoylsalicin, acetylcinnamoylsalicortin, and HCH-cinnamoylsalicortin isomers] from compounds with a benzoyl group (tremulacin, tremuloidin, acetyltremulacin, and HCH-tremulacin). The second component (PC2) separated compounds with an 2′-acetyl, (2′-acetylsalicin, 2′-acetylsalicortin, and lasiandrin) from those with an additional HCH group (HCH-salicortin, HCH-tremulacin, and HCH-cinnamoylsalicortin).

### Chemotype and environment differences, main salicinoid correlations, and tree origin

**Figure 3:**
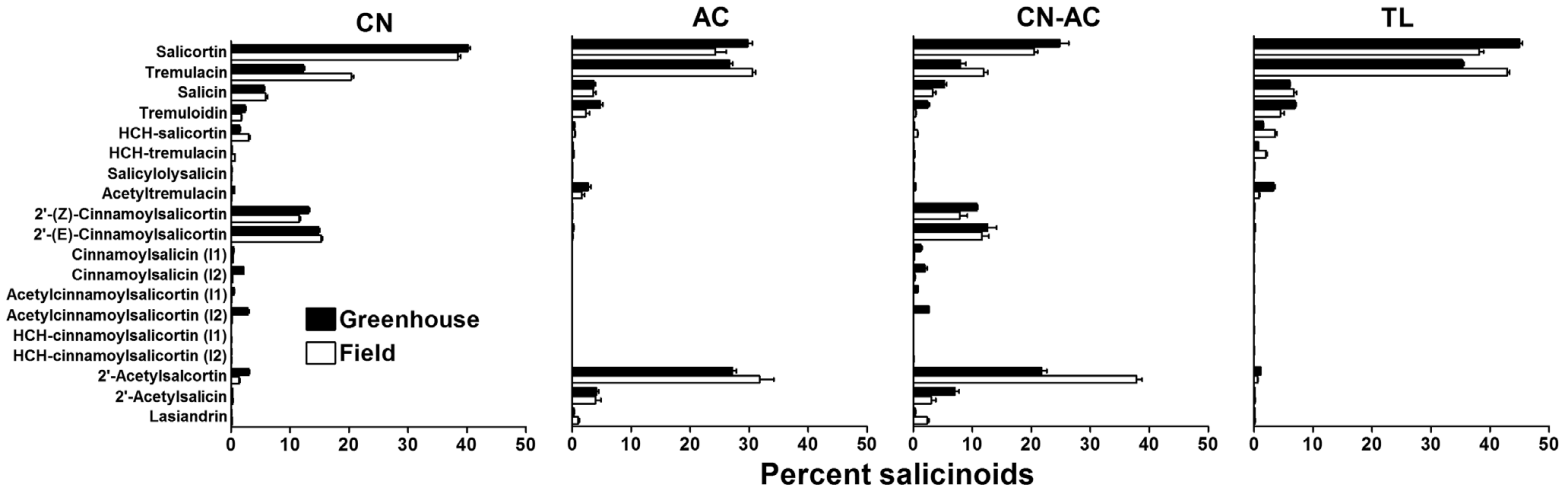
Percentages (± SE) of 19 salicinoids from greenhouse (solid bars) and field (open bars) grown *Populus tremula* trees from the SwAsp collection of the four identified chemotypes, CN (2′-cinnamoyl), AC (2′-acetyl), CN-AC (2′-cinnamoyl/2′-acetyl), and TL (*tremuloides*-like)]. I1 and I2 =  isomer 1 and 2, respectively, based upon UHPLC retention times.

The two-factor MANOVA test showed that salicinoid profile differed for both environment (*Wilks' λ* = 0.32, *F*_19, 292_ = 32.3, *P*<0.001), chemotype (*Wilks' λ* = 0.001, *F*_57, 871_ = 154.5, *P*<0.001), and their interaction (*Wilks' λ* = 0.09, *F*_57, 871_ = 18.5, *P*<0.001; Fig. 3). Individual ANOVA results showed that all salicinoids differed with chemotype, and all compounds, except salicin, 2′-(*E*)-cinnamoylsalicortin, isomer 2 of HCH-cinnamoylsalicortin, and 2′-acetylsalicin, also differed between environment (Fig. 3; Table 2). In general, greenhouse grown trees contained more salicortin, tremuloidin, acetyltremulacin, and 2′-(*Z*)-cinnamoylsalicortin, and field trees contained more tremulacin. For isomer 2 of HCH-cinnamoylsalicortin the difference between environments was only apparent due to the interaction of environment with chemotype. Many other salicinoids interacted between these two factors, usually due to differing ratios of particular salicinoids in samples of the different chemotypes, as in the case of both salicortin and tremulacin. In some instances, the compound patterns were reversed between chemotypes. For instance, AC and CN-AC chemotypes had much more 2'-acetylsalicortin in field grown trees.

**Table 2:** *F* and *P* values from the two-factor ANOVA comparing percentages of individual leaf extract salicinoids from *Populus tremula* trees of four chemotypes (CN, AC, CN-AC, and TL), grown in two environments (greenhouse and field), and their interaction (E*Ct).

Compound	Environment		Chemotype		E*Ct	
	*df* = 1		*df* = 3		*df* = 3	
	*F*	*P*	*F*	*P*	*F*	*P*
Salicortin	17.0	<0.001	90.1	<0.001	8.2	<0.001
Tremulacin	60.5	<0.001	436.8	<0.001	3.8	0.011
Salicin	0.4	0.508	10.0	<0.001	0.8	0.497
Tremuloidin	22.6	<0.001	71.8	<0.001	3.8	0.010
HCH-salicortin	10.9	0.001	22.6	<0.001	2.3	0.081
HCH-tremulacin	25.8	<0.001	67.2	<0.001	1.1	0.335
Salicyloylsalicin	26.9	<0.001	14.8	<0.001	2.4	0.066
Acetyltremulacin	22.0	<0.001	35.2	<0.001	1.5	0.225
2'-(*Z*)-Cinnamoylsalicortin	4.9	0.028	28.5	<0.001	4.5	0.004
2'-(*E*)-Cinnamoylsalicortin	0.2	0.645	456.4	<0.001	0.9	0.458
Cinnamoylsalicortin (*I*1)	17.4	<0.001	282.1	<0.001	6.0	0.001
Cinnamoylsalicortin (*I*2)	178.2	<0.001	317.2	<0.001	288.7	<0.001
Acetylcinnamoylsalicortin (*I*1)	14.9	0.000	21.3	<0.001	16.7	<0.001
Acetylcinnamoylsalicortin (*I*2)	17.4	<0.001	38.7	<0.001	30.5	<0.001
HCH-cinnamoylsalicortin (*I*1)	7.6	0.006	607.4	<0.001	21.9	<0.001
HCH-cinnamoylsalicortin (*I*2)	3.2	0.073	855.5	<0.001	2.7	0.048
2'-Acetylsalcortin	17.0	<0.001	90.1	<0.001	8.2	<0.001
2'-Acetylsalicin	2.4	0.126	123.5	<0.001	1.5	0.223
Lasiandrin	18.8	<0.001	27.2	<0.001	2.7	0.048

*I*1 and *I*2 indicate isomers 1 and 2, respectively, designated by UHPLC retention times. See Figure 3 for corresponding data.

**Figure 4:**
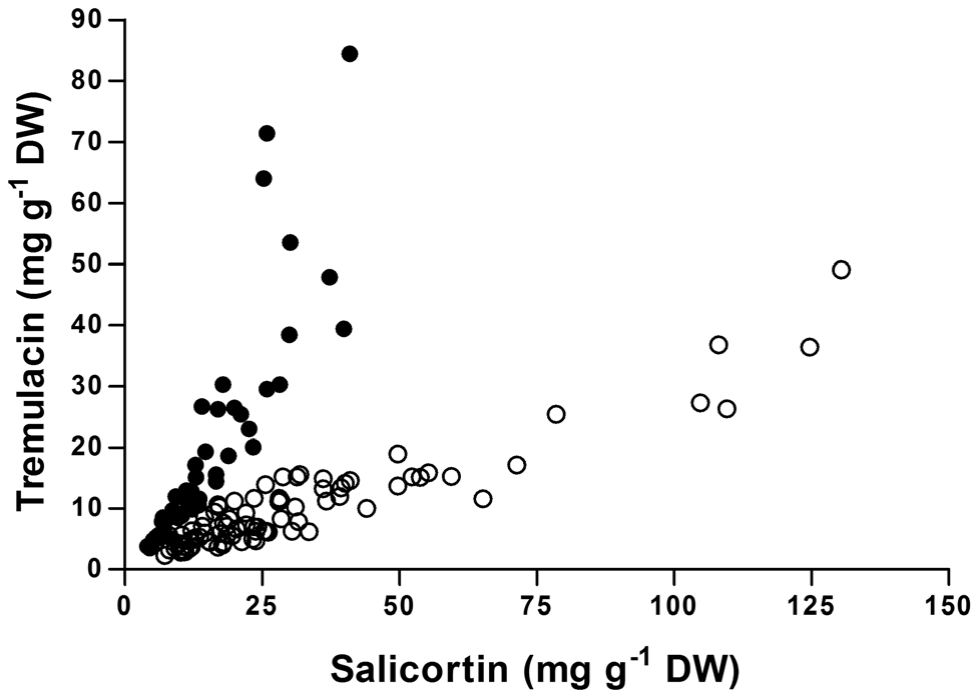
Relationship between the two most abundant salicinoids, tremulacin and salicortin, in field grown *Populus tremula* trees for chemotypes low (TL; solid circles) or high (CN; open circles) in 2′-cinnamoylsalicortin.

Correlations between the amounts of tremulacin and salicortin in field grown individuals with either low (TL chemotypes; *r* = 0.87, *P*<0.001, *N* = 49) or high (CN chemotypes; *r* = 0.93, *P*<0.001, *N* = 83) levels of 2′-cinnamoylsalicortin showed strong positive relationships in both cases (Fig. 4). The patterns were, however, notably different with CN trees containing considerably less tremulacin with increasing amounts of salicortin (Fisher's *Z* = 1.71, *P*<0.05). The relative amounts of these two salicinoids varied with chemotype from an approximately even relationship of 1 tremulacin: 1 salicortin (in mg g^-1^) in TL chemotypes to a 1∶4 relationship in the CN chemotype.

**Figure 5:**
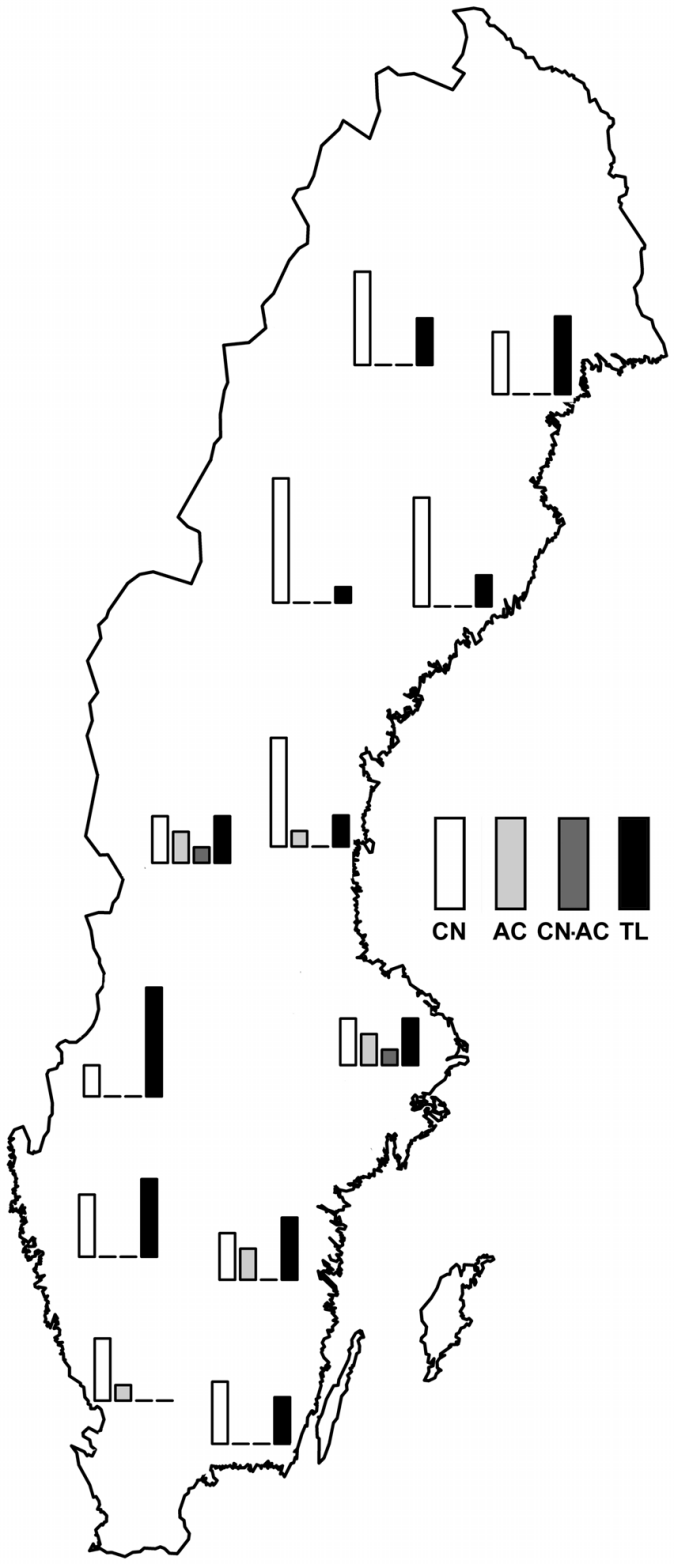
Salicinoid chemotypes of *Populus tremula* trees (the SwAsp collection) collected from ten locations throughout Sweden. White  =  CN (2′-cinnamoyl), light gray  =  AC (2′-acetyl), dark gray  =  CN-AC (2′-cinnamoyl/2′-acetyl), and black  =  TL (*tremuloides*-like). Bar height corresponds to the number of individuals of each chemotype.

Trees belonging to the CN and TL chemotypes occurred at sites throughout Sweden (Fig. 5) and their distribution did not follow a simple geographic or clinal pattern. The genotypes containing 2′-acetyl compounds (AC and CN-AC) originated from central and southern Sweden.

### Clonal repeatability of salicinoids

Overall, clonal repeatabilities (*H* ^2^; broad-sense heritability) for salicinoids in this study were high with the greenhouse population showing slightly higher values compared to field grown trees (Table 3). The most notable examples that contributed to this trend were salicortin, tremuloidin, and the isomer pairs of cinnamoylsalicin and acetylcinnamoylsalicortin. Both acetyltremulacin and lasiandrin had substantially lower clonal repeatabilities in the greenhouse environment than in the field. In addition, all salicinoids with higher clonal repeatabilities in the field contained two HCH moieties.

**Table 3:** Clonal repeatabilities (*H* ^2^) of 19 salicinoids from 85 clones of *Populus tremula* from greenhouse (GH), field, and combined (All) populations. *I*1 and *I*2 indicate isomers 1 and 2, designated by UHPLC retention times.

Compound	*H* ^2^		
	GH	Field	All
Salicortin	0.87	0.70	0.67
Tremulacin	0.98	0.98	0.92
Salicin	0.46	0.45	0.29
Tremuloidin	0.81	0.59	0.60
HCH-salicortin	0.86	0.91	0.76
HCH-tremulacin	0.85	0.96	0.80
Salicyloylsalicin	0.90	0.84	0.69
Acetyltremulacin	0.95	0.90	0.83
2′-(*Z*)-Cinnamoylsalicortin	0.98	0.96	0.96
2′-(*E*)-Cinnamoylsalicortin	0.99	0.98	0.98
Cinnamoylsalicin (*I*1)	0.79	0.41	0.54
Cinnamoylsalicin (*I*2)	0.94	0.74	0.57
Acetylcinnamoylsalicortin (*I*1)	0.88	0.77	0.51
Acetylcinnamoylsalicortin (*I*2)	0.92	0.75	0.54
HCH-cinnamoylsalicortin (*I*1)	0.88	0.87	0.70
HCH-cinnamoylsalicortin (*I*2)	0.86	0.91	0.83
2′-Acetylsalicortin	0.99	0.99	0.98
2′-Acetylsalicin	0.96	0.95	0.92
Lasiandrin	0.76	0.89	0.73

## Discussion

### Salicinoid survey of *P. tremula*

On the basis of literature studies (i.e., [3]), previous salicinoid analyses [29], [39], and theoretical structures, we identified a total of 19 potentially bioactive salicinoid compounds from *P. tremula*, adding nine new structures to those already described by Abreu et al. [29].

Many of these salicinoids are dominant or characteristic for other species in the Salicaceae [3]. The acetylated compounds 2′-acetylsalicin, 2′-acetylsalicortin, lasiandrin, and acetyltremulacin co-occur in *Salix pentandra* and *S. lasiandra* [35], [47], [48]. HCH-salicortin (salicortin derivative or disalicortin [49]) and HCH-tremulacin both occur in *S. myrsinifolia* [2], [49]. HCH-salicortin was also isolated from *P. fremontii* and its F1 hybrids with *P. angustifolia* [30], and studies with *S. sericea* found 2′-(*E*)-cinnamoylsalicortin [31].

### Frequencies and distribution of key compounds in SwAsp

Salicinoid profiles readily divided SwAsp genotypes into four chemotypes, based upon the presence or absence of specific moieties. The 2′-cinnamoyl moiety defined the most abundant chemotype with 53 percent representation in SwAsp. In general, the capability to synthesize cinnamoyl salicinoids appears to be mostly either present or absent in a genotype and thus accounts for the strongest division of the population. The ability to add acetyl moieties appeared less channeled compared to the cinnamoyl addition, but curiously acetylcinnamoylsalicortin had somewhat higher clonal repeatabilities in the less stable field environment (Table 3). Across SwAsp chemotypes we found salicortin, tremulacin, 2′-acetylsalicortin, and the 2′-cinnamoylsalicortins as the dominant salicinoids and when present they usually occurred in relatively high amounts.

The distribution of SwAsp chemotypes throughout Sweden did not resemble the clinal structure reported for phenological traits [36], or the north-south clustering that characterizes inducible defense genes [50]. The two most abundant chemotype groups (CN and TL) were evenly distributed among collection sites, whereas the AC and CN-AC chemotypes mainly originated from the central and southern part of the country. After the last ice age, many plants and animals invaded Sweden from both the north east and the south west and later united in central Sweden (along Limes Norrlandicus) where they either formed hybrid zones or distinct subpopulations [51]. Although the postglacial invasion of aspen in Sweden had weak effects on the genetic differentiation of neutral markers [52], it could have had local effects on adaptive traits [40]. Introgression of defense associated genes naturally occurs in hybrid zones [53], [54], and hybrid zones and admixture populations may also promote novel genotypes [30], [32], [33], [53], [54]. Evidence of salicinoid inheritance from hybrid zones includes additive inheritance of HCH-salicortin by F_1_ hybrids of *P. fremontii* and *P. angustifolia* from the *P. fremontii* parent [30]. Similarly, additive inheritance was found in a *P. tremula-alba* hybrid zone for HCH-salicortin and 2′-acetylsalicortin, whereas HCH-tremulacin was trangressive at higher concentrations [33]. The geographical distribution of the AC and CN-AC SwAsp chemotypes in central and southern Sweden could consequently mirror fitness properties, recent evolution, or introgression.

### Elusive salicinoid biosynthesis

Tsai et al. [16] proposed salicin as the substrate for salicinoid biosynthesis, but studies using labeled compounds could not confirm this suggestion [21] and the biosynthetic route of salicinoids remains poorly understood [15], [16], [21], [22]. Confirming the work by Abreu et al. [29], we found strong correlations between amounts of 2′-acetylsalicin and 2′-acetylsalicortin, as well as associations between cinnamoyl-containing salicinoids. The dynamics of salicinoid pools thus appear to depend on the presence or absence of characteristic chemotype moieties and the relative abundance of common salicinoids vary between chemotypes. For example, the concentration of tremulacin is generally lower in CN chemotype individuals compared to the TL chemotype (Fig. 4). This may reflect competition for salicortin as a substrate for addition of either a cinnamoyl or benzoyl group for synthesis of 2′-cinnamoylsalicortin or tremulacin, respectively.

The composite structure of the salicinoids could further suggest that relatively few enzymes are involved in their biosynthesis. Keeling and Bohlmann [26] and Degenhardt et al. [25] have demonstrated how a few key genes that are differently but consistently expressed result in unique terpenoid profiles in individual conifers, creating high terpenoid diversity at the population level. Although at a lower diversity, the salicinoids of SwAsp may be biosynthesized according to a similar strategy. Association studies relate specific traits to genetic patterns [55], and could potentially be a promising way to get insight into the salicinoid biosynthesis. Thus, rather than relating single compounds to gene sequences, association studies may benefit from grouping the compounds on the basis of presence and absence of specific salicinoid moieties (see also [54]).

### Salicinoid profile stability and implications for chemical ecology

Salicinoid composition of individual trees across environments with very different growth histories showed overall high heritabilities, confirming that both salicinoid quality (composition) and quantity (abundance) is likely to be highly channeled in aspen, and thus relatively stable in different environments [29], [56]. Interestingly, we found that the most represented group in SwAsp, the CN chemotype, also showed the largest plasticity of salicinoids in response to environmental differences. This suggests an elevated level of plasticity in cinnamoyl-containing salicinoid expression and a potential fitness advantage. Salicinoid toxicity to herbivores has been attributed to the HCH moiety, even at low concentrations [3], [14], [57]. Most of the newly described salicinoids in this study contain one or two HCH groups, and the high heritability of the HCH containing compounds in field samples supports that they may be emphasized in more challenging environments (Table 3). Lindroth et al. [14] found that tremulacin greatly reduced herbivore survival and performance and suggested that the benzoyl group synergizes the toxic effect of the HCH group. Similarly, the cinnamoyl and acetyl groups may also synergize the effects of the HCH moiety on relevant molecules (2′-cinnamoylsalicortin and 2′-acetylsalicortin).

Given the apparent stability of salicinoid profiles in *P. tremula* and the clearly defined chemotypes, the SwAsp collection represents an ideal system for the study of chemical-ecological interactions. With almost the entire collection in tissue culture, and with the present robust salicinoid profiling (Material S2), we can propagate our chemotypes to specifically test properties of resistance and tolerance to various kinds of associated herbivores and fungi [13], [45]. In conclusion, we observed a striking division of aspen into four chemotypes, some of which co-occur across latitudes. These chemotypes differ in the dominant moieties and future studies are needed to explore their relative bioactivity, especially including the new dominant compounds (2′-cinnamoylsalicortins and 2′-acetylsalicortin) that are poorly studied. We further suggest that ecological studies, both locally and across latitudes, must take *P. tremula's* salicinoid diversity into account.

## Supporting Information

Material S1 Salicinoid identification: Exact masses of 55 salicinoid compounds (**Table S1**), Literature references (**List S1**), UV spectra of nine new salicinoids found in the *P. tremula* foliage (**Fig. S1** ***a***), and high-resolution MS/MS (**Fig. S1** ***b***).
(DOCX)

Material S2 Average percentages of 19 salicinoids from the foliage of different *Populus tremula* clones (Clone), grown in two different environments (Evir: GH  =  greenhouse, Sävar  =  field) and mg g^−1^ for field trees. Chemo  =  chemotype: CN  =  2′-cinnamoyl, AC  =  2′- acetyl, CN-AC  =  2′-cinnamoyl/2′-acetyl, and TL  =  *tremuloides*-like. Salicinoids: 1 =  salicortin, 2 =  tremulacin, 3 =  salicin, 4 =  tremuloidin, 5 =  HCH-salicortin, 6 =  HCH-tremulacin, 7 =  salicyloylsalicin, 8 =  6′-acetyl-tremulacin, 9 =  2′-(*Z*)-cinnamoylsalicortin, 10 =  2′-(*E*)- cinnamoylsalicortin, 11 =  cinnamoylsalicin I*1*, 12 =  cinnamoylsalicin I*2*, 13 =  acetylcinnamoylsalicortin I*1*, 14 =  acetylcinnamoylsalicortin I*2*, 15 =  HCH-cinnamoylsalicortin I*1*, 16 =  HCH-cinnamoylsalicortin I*2*, 17 =  2′-acetylsalicortin, 18 =  2′-acetylsalicin, 19 =  lasiandrin (HCH-2'-acetylsalicortin). I*1* and I*2* =  isomers 1 and 2, respectively.
(DOCX)
