# Supplementary material for: No Evidence of Geographical Structure of Salicinoid Chemotypes within Populus Tremula
Source: PLoS One. 2014 Oct 9;9(10):e107189. doi: 10.1371/journal.pone.0107189 (PMC4191948; doi:10.1371/journal.pone.0107189)

**Supporting Material S1.** Salicinoid identification: Exact masses of 55 salicinoid compounds (**Table S1**), References (**List S1**), UV spectra of nine new salicinoids found in the *P. tremula* foliage (**Fig. S1*a***) and high-resolution MS/MS (**Fig. S1*b***)

**Table S1*a*.** Monoisotopic ion exact masses of 55 known (numbers in brackets refer to List S1) and theoretical (*) salicinoids searched for in *P. tremula* UHPLC-TOF/MS chromatograms in this study. Compounds in bold were found in *P. tremula* (Information from this study and references [2,8] in List S1, see below).

**Compound**

**Molecular**

**Monoisotopic exact mass**

**Reference**

|  | **formula** | **[M]** | **[M-H]- [M+FA-H]-** | | |
| --- | --- | --- | --- | --- | --- |
| **Salicin** | C13H18O7 | 286.1052 | 285.0980 | 331.1034 | 1, 2 |
| **2'-Acetylsalicin** | C15H20O8 | 328.1158 | 327.1085 | 373.1140 | 1, 2 |
| Fragilin | C15H20O8 | 328.1158 | 327.1085 | 373.1140 | 1 |
| Diacetylsalicin | C17H22O9 | 370.1264 | 369.1191 | 415.1246 | * |
| **Tremuloidin** | C20H22O8 | 390.1315 | 389.1242 | 435.1297 | 1, 2 |
| Chaenomeloidin | C20H22O8 | 390.1315 | 389.1242 | 435.1297 | 1 |
| Populin | C20H22O8 | 390.1315 | 389.1242 | 435.1297 | 1 |
| Deltoidin | C20H22O9 | 406.1264 | 405.1191 | 451.1246 | 1 |
| Nigracin | C20H22O9 | 406.1264 | 405.1191 | 451.1246 | 1 |
| **Salicyloylsalicin** | C20H22O9 | 406.1264 | 405.1191 | 451.1246 | 1 |
| Salireposide | C20H22O9 | 406.1264 | 405.1191 | 451.1246 | 1 |
| Trichocarpin | C20H22O9 | 406.1264 | 405.1191 | 451.1246 | 3 |
| **Cinnamoylsalicin** | C22H24O8 | 416.1471 | 415.1398 | 461.1453 | * |
| **Salicortin** | C20H24O10 | 424.1369 | 423.1297 | 469.1351 | 1, 2 |
| Populoside B | C22H24O9 | 432.1420 | 431.1347 | 477.1402 | 1 |
| Trichocarposide | C22H24O9 | 432.1420 | 431.1347 | 477.1402 | 1 |
| Grandidentanin | C21H28O9 | 432.1420 | 431.1347 | 477.1402 | 4 |
| Grandidentoside | C21H28O10 | 440.1683 | 439.1610 | 485.1665 | 5 |
| Populoside | C22H24O10 | 448.1369 | 447.1297 | 493.1351 | 1 |
| Populoside A | C22H24O10 | 448.1369 | 447.1297 | 493.1351 | 1 |
| Acetylsalicyloylsalicin | C22H24O10 | 448.1369 | 447.1296 | 493.1351 | * |
| Diglucoside salicin | C19H28O12 | 448.1581 | 447.1508 | 493.1563 | 6 |
| Acetylcinnamoylsalicin | C24H26O9 | 458.1577 | 457.1504 | 503.1559 | * |
| Populoside C | C23H26O10 | 462.1526 | 461.1453 | 507.1508 | 1 |

| **2'-Acetylsalicortin** | C22H26O11 | 466.1475 | 465.1402 | 511.1457 | 1 |
| --- | --- | --- | --- | --- | --- |
| Diacetylsalicyloylsalicin | C24H26O11 | 490.1475 | 489.1402 | 535.1457 | * |
| 6'-Benzoyltremuloidin | C27H26O9 | 494.1577 | 493.1504 | 539.1559 | * |
| Diacetylsalicortin | C24H28O12 | 508.1581 | 507.1508 | 553.1563 | 7 |
| Salicyloyltremuloidin | C27H26O10 | 510.1526 | 509.1453 | 555.1508 | 1 |
| 6’-Benzoylcinnamoylsalicin | C29H28O9 | 520.1733 | 519.1660 | 565.1715 | * |
| 6'-Cinnamoyltremuloidin | C29H28O9 | 520.1733 | 519.1660 | 565.1715 | * |
| **Tremulacin** | C27H28O11 | 528.1632 | 527.1559 | 573.1614 | 1, 2 |
| HCH-tremuloidin | C27H28O11 | 528.1632 | 527.1559 | 573.1614 | * |
| Cinnamoylsalicyloylsalicin | C29H28O10 | 536.1683 | 535.1610 | 581.1665 | * |
| 2-Hydroxybenzoylsalicortin | C27H28O12 | 544.1581 | 543.1508 | 589.1563 | * |
| HCH-deltoidin | C27H28O12 | 544.1581 | 543.1508 | 589.1563 | * |
| HCH-Salicyloylsalicin | C27H28O12 | 544.1581 | 543.1508 | 589.1563 | * |
| HCH-nigracin (2’-Bz) | C27H28O12 | 544.1581 | 543.1508 | 589.1563 | * |
| HCH-nigracin (6’-Bz) | C27H28O12 | 544.1581 | 543.1508 | 589.1563 | * |
| Dicinnamoylsalicin | C31H30O9 | 546.1890 | 545.1817 | 591.1872 | * |
| Acetylsalicyloyltremuloidin | C29H28O11 | 552.1632 | 551.1559 | 597.1614 | * |
| **2’-(*Z*)-Cinnamoylsalicortin** | C29H30O11 | 554.1788 | 553.1715 | 599.1770 | 8 |
| **2’-(*E*)-Cinnamoylsalicortin** | C29H30O11 | 554.1788 | 553.1715 | 599.1770 | 1, 2 |
| **HCH-salicortin** | C27H30O13 | 562.1686 | 561.1614 | 607.1668 | 1 |
| **6'-Acetyltremulacin** | C29H30O12 | 570.1737 | 569.1664 | 615.1719 | 2 |
| HCH-acetylsalicyloylsalicin | C29H30O13 | 586.1686 | 585.1613 | 631.1668 | * |
| **Acetylcinnamoylsalicortin** | C31H32O12 | 596.1894 | 595.1821 | 641.1876 | * |
| **Lasiandrin** | C29H32O14 | 604.1792 | 603.1719 | 649.1774 | 1 |
| 6'-Benzoyltremulacin | C34H32O12 | 632.1894 | 631.1821 | 677.1876 | * |
| HCH-Salicyloyltremuloidin | C34H32O13 | 648.1843 | 647.1770 | 693.1825 | * |
| 6'-Cinnamoyltremulacin | C36H34O12 | 658.2050 | 657.1977 | 703.2032 | * |
| **HCH-tremulacin** | C34H34O14 | 666.1949 | 665.1876 | 711.1931 | 9 |
| Dicinnamoylsalicyloylsalicin | C38H34O11 | 666.2101 | 665.2028 | 711.2083 | * |
| Dicinnamoylsalicortin | C38H36O12 | 684.2207 | 683.2134 | 729.2189 | * |
| **HCH-cinnamoylsalicortin** | C36H36O14 | 692.2105 | 691.2032 | 737.2087 | * |

**Reference List S1.**

1. **Boeckler GA, Gershenzon J, Unsicker SB. 2011.** Phenolic glycosides of the Salicaceae and their role as anti-herbivore defenses. *Phytochemistry* **72**(13): 1497-1509.

2. **Abreu IN, Ahnlund M, Moritz T, Albrectsen BR. 2011.** UHPLC-ESI/TOFMS

determination of salicylate-like phenolic glycosides in *Populus tremula* leaves. *Journal of*

*Chemical Ecology* **37**(8): 857-870.

4. **Si CL, Xu J, Kim JK, Bae YS, Liu PT, Liu Z. 2011.** Antioxidant properties and structural analysis of phenolic glucosides from bark of *Populus ussuriensis* Kom. *Wood Science and Technology* **45**(1): 5-13.

5. **Erickson RL, Pearl IA, Darling SF. 1970.** Populoside and grandidentoside from bark of

*Populus grandidentata*. *Phytochemistry* **9**(4): 857-863.

6. **Ruuhola TM, Sipura M, Nousiainen O, Tahvanainen J. 2001.** Systemic induction of salicylates in *Salix myrsinifolia* (Salisb.). *Annals of Botany* **88**(3): 483-497.

7. **Ruuhola T, Julkunen-Tiitto R. 2003.** Trade-off between synthesis of salicylates and growth of micropropagated *Salix pentandra*. *Journal of Chemical Ecology* **29**(7): 1565-1588

8. **Keefover-Ring K, Carlsson M, Albrectsen BR. 2014.** 2’-(*Z*)-Cinnamoylsalicortin: A novel salicinoid isolated from *Populus tremula*. *Phytochemistry Letters* **7**: 212-216.

9. **Paunonen R, Julkunen-Tiitto R, Tegelberg R, Rousi M, Heiska S. 2009.** Salicylate and biomass yield, and leaf phenolics of dark-leaved willow (*Salix myrsinifolia* Salisb.) clones under different cultivation methods after the second cultivation cycle. *Industrial Crops and Products* **29**(2-3): 261-268.

**Fig. S1*a*.** UV spectra (210-400 nm) of the new salicinoids found in the foliage of *Populus tremula*. See Materials and Methods for instrument conditions. Numbers on spectra indicate λmax

**HCH-salicortin**


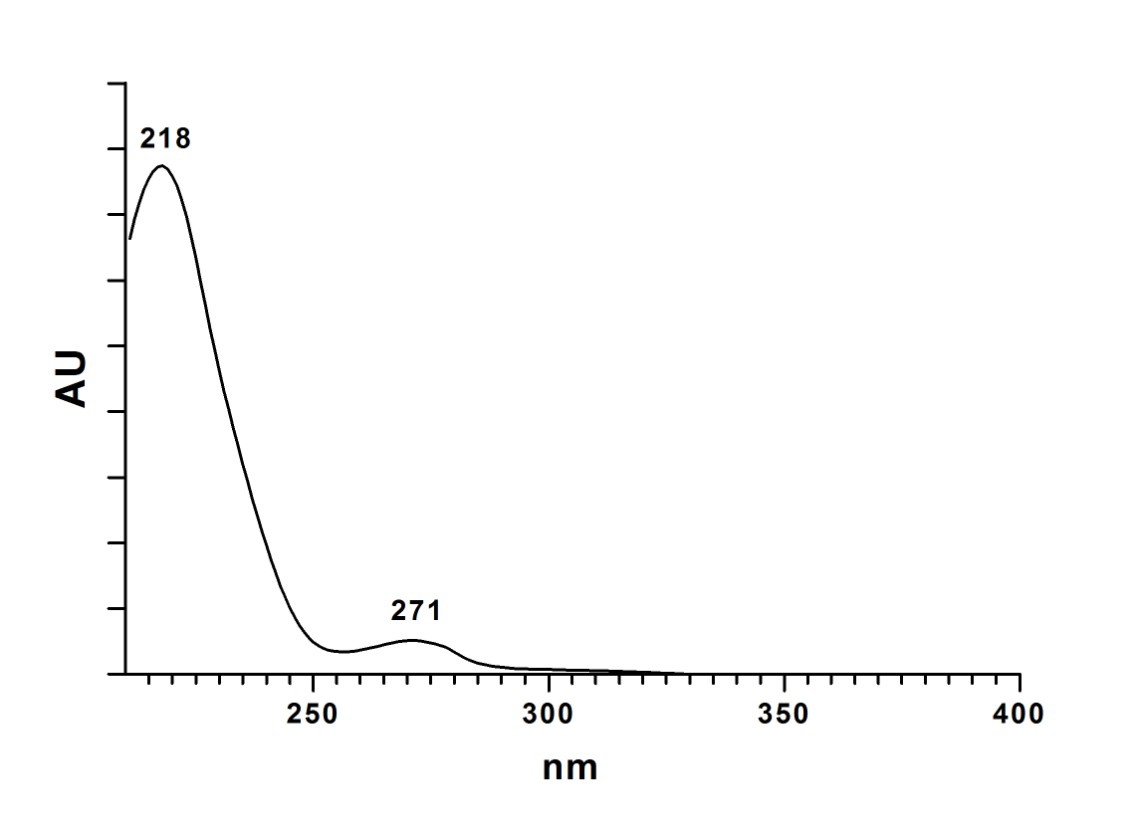


**HCH-tremulacin**


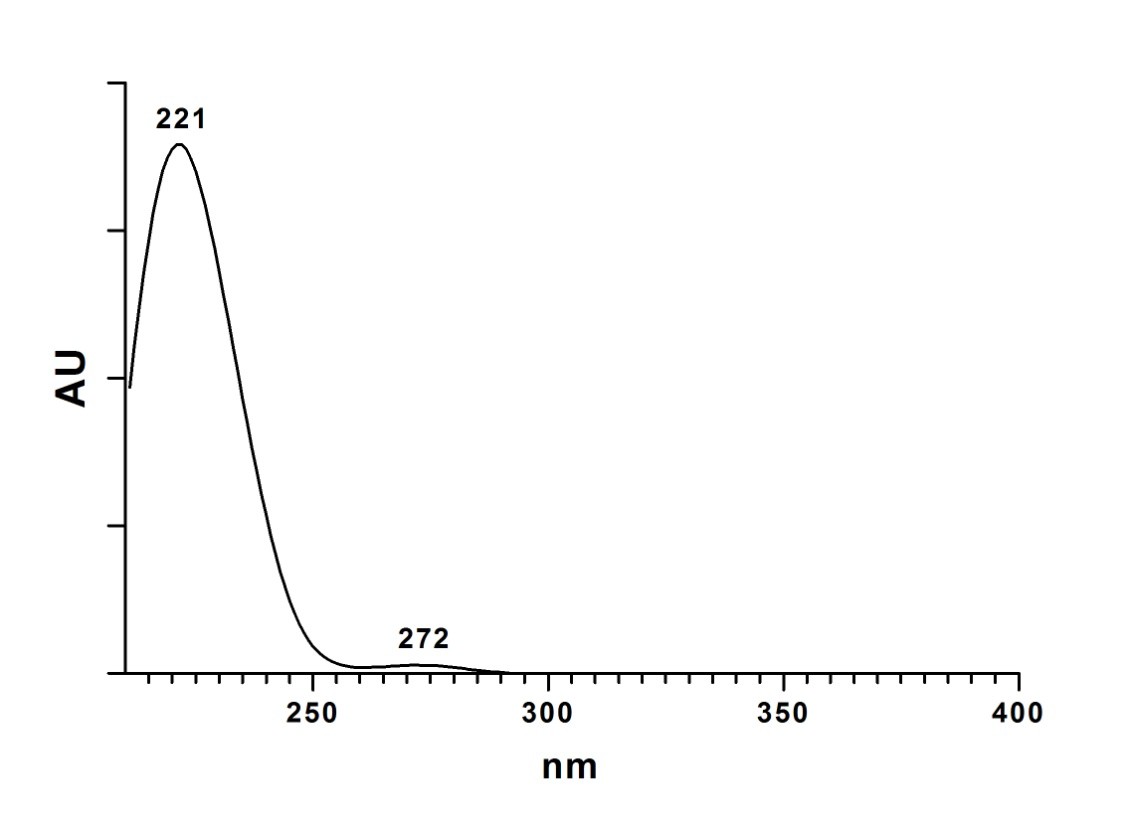


**Cinnamoylsalicin isomers 1 (top) and 2 (bottom)**


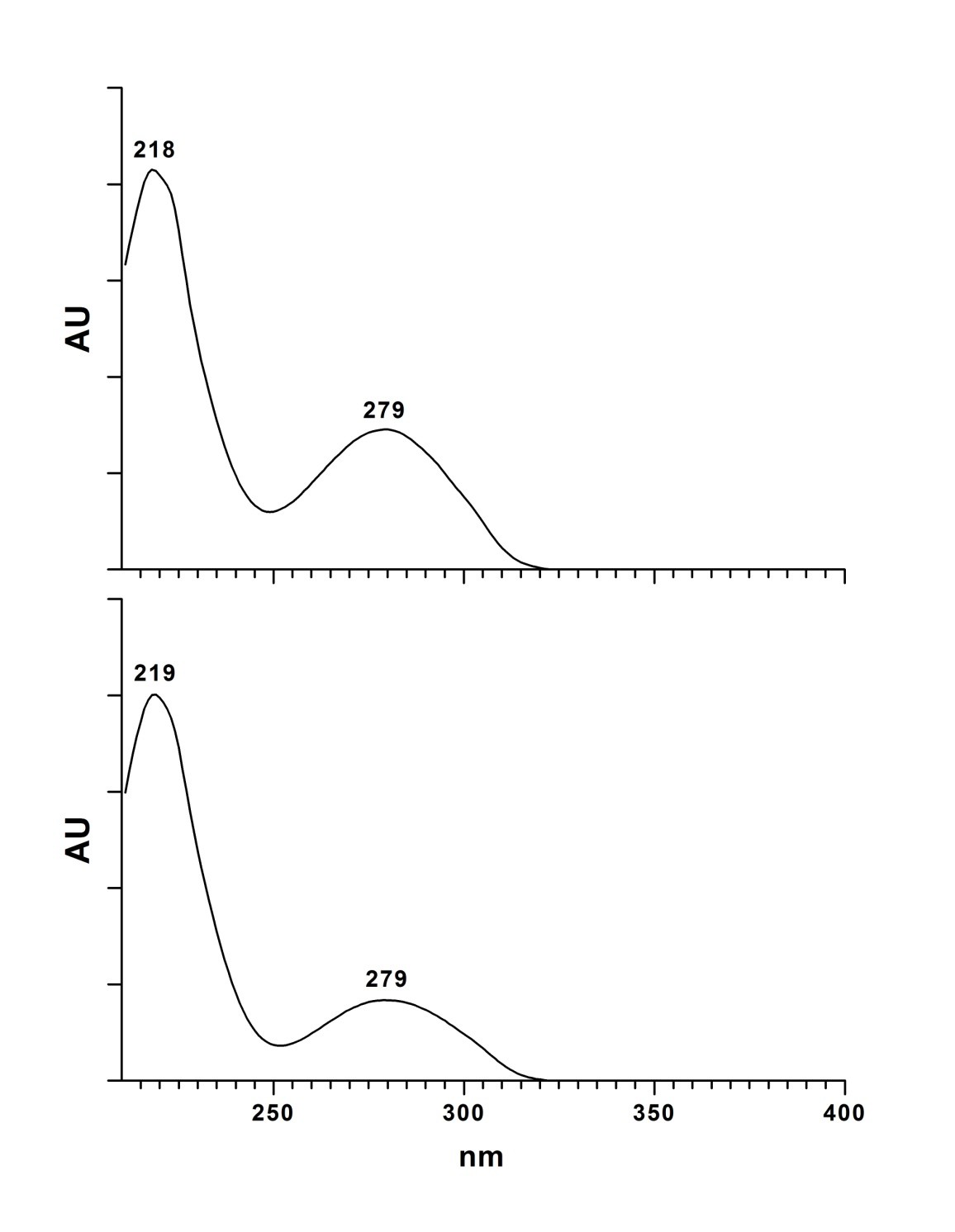


**Acetylcinnamoylsalicortin isomers 1 (top) and 2 (bottom)**


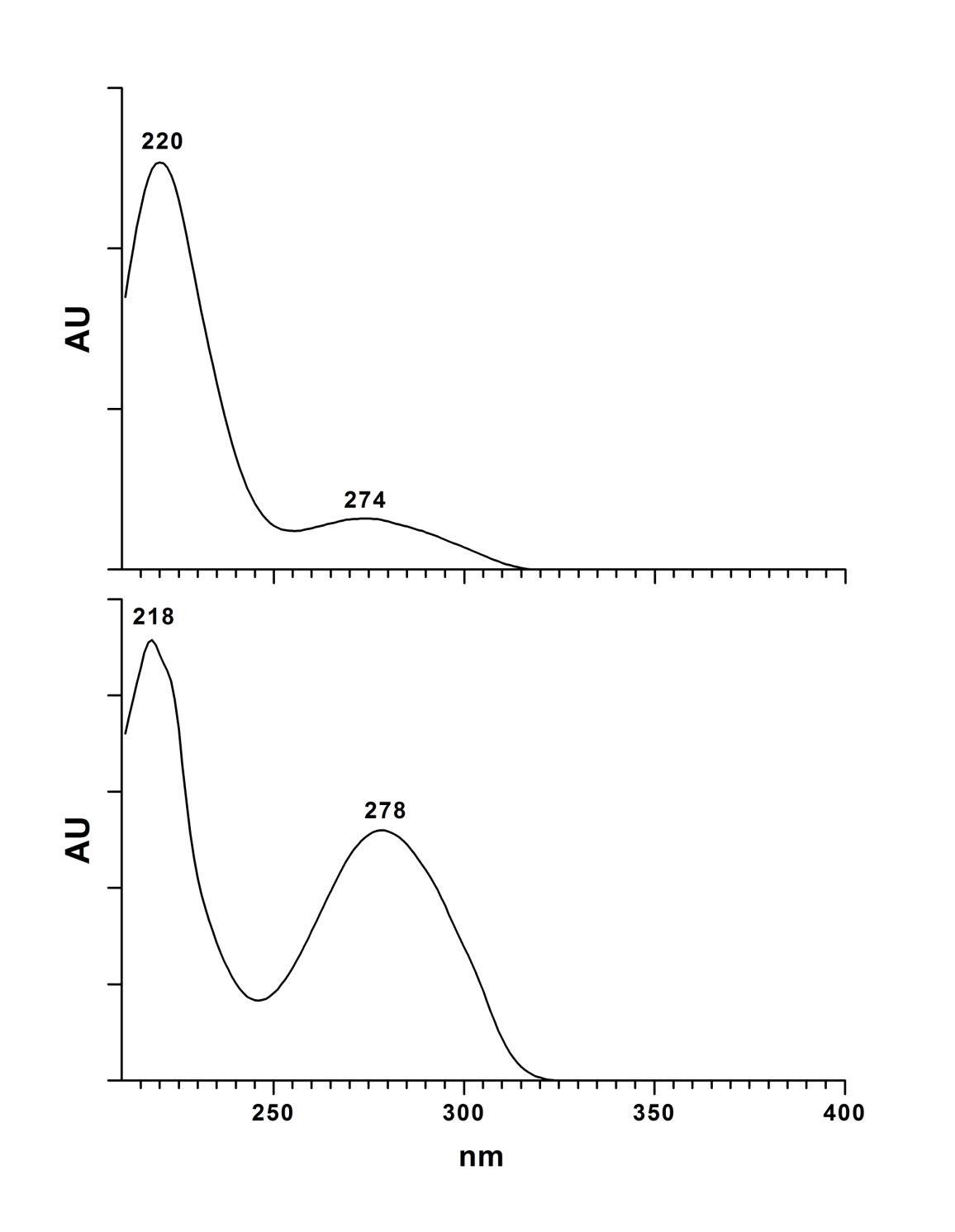


**HCH-cinnamoylsalicortin isomers 1 (top) and 2 (bottom)**


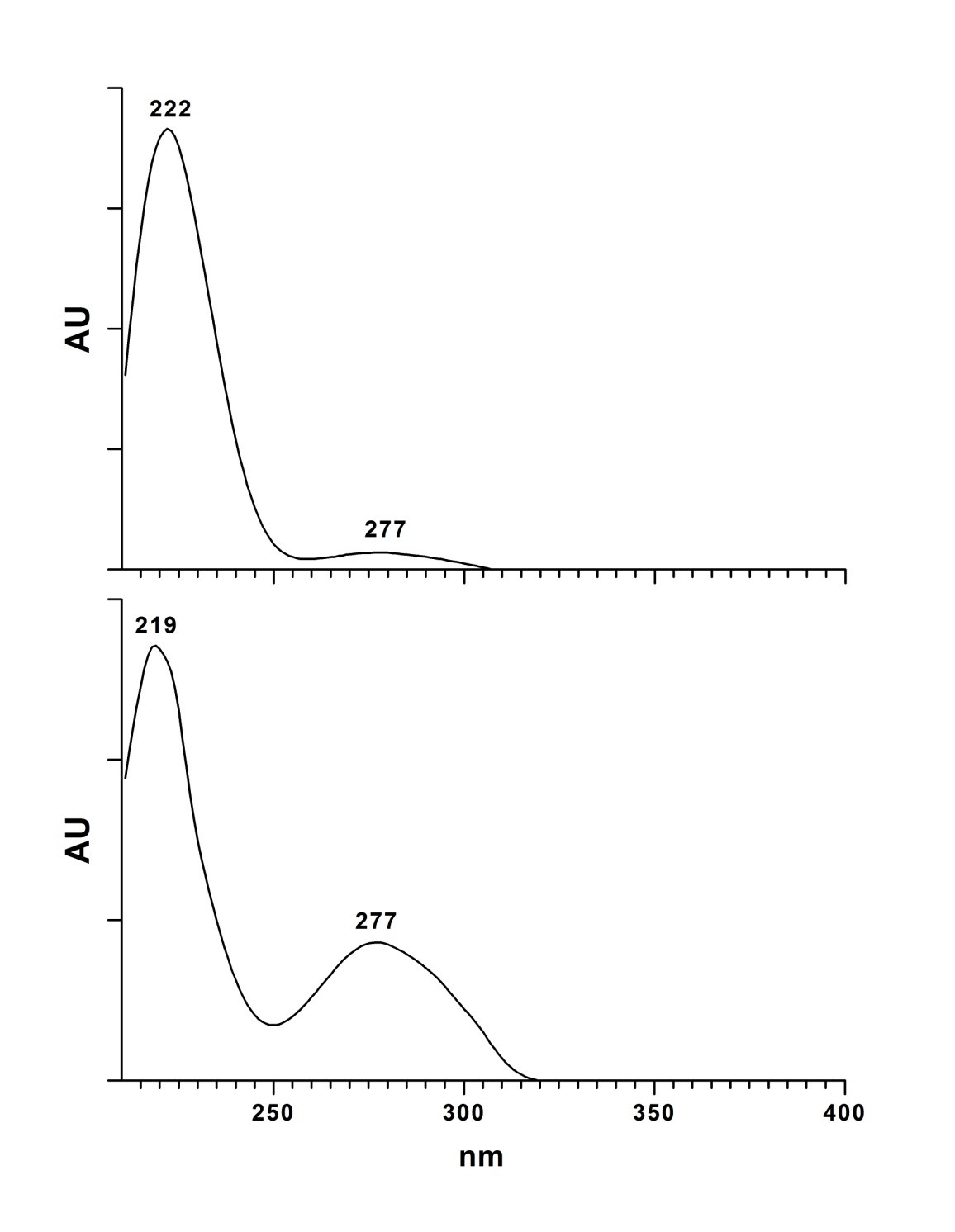


**Lasiandrin (HCH-2’-acetylsalicortin)**


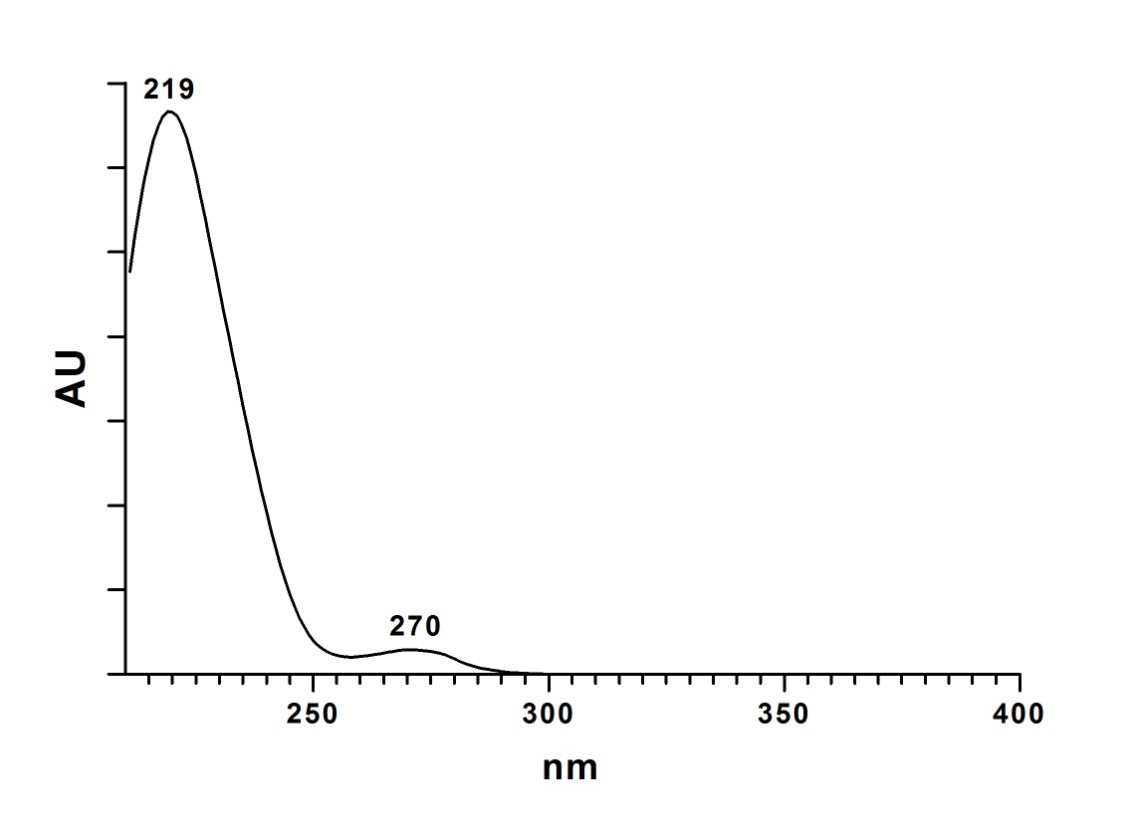


**Fig. S1*b*** High-resolution MS/MS spectra of the new salicinoids found in the foliage of *Populus tremula*. See Methods and Materials for instrument conditions. Arrows on structures show the most abundant fragments. See **Table 1** for a list of fragments and their relative intensities

**HCH-salicortin** - Used [M-H]- *m*/*z* 561.161 for fragmentation


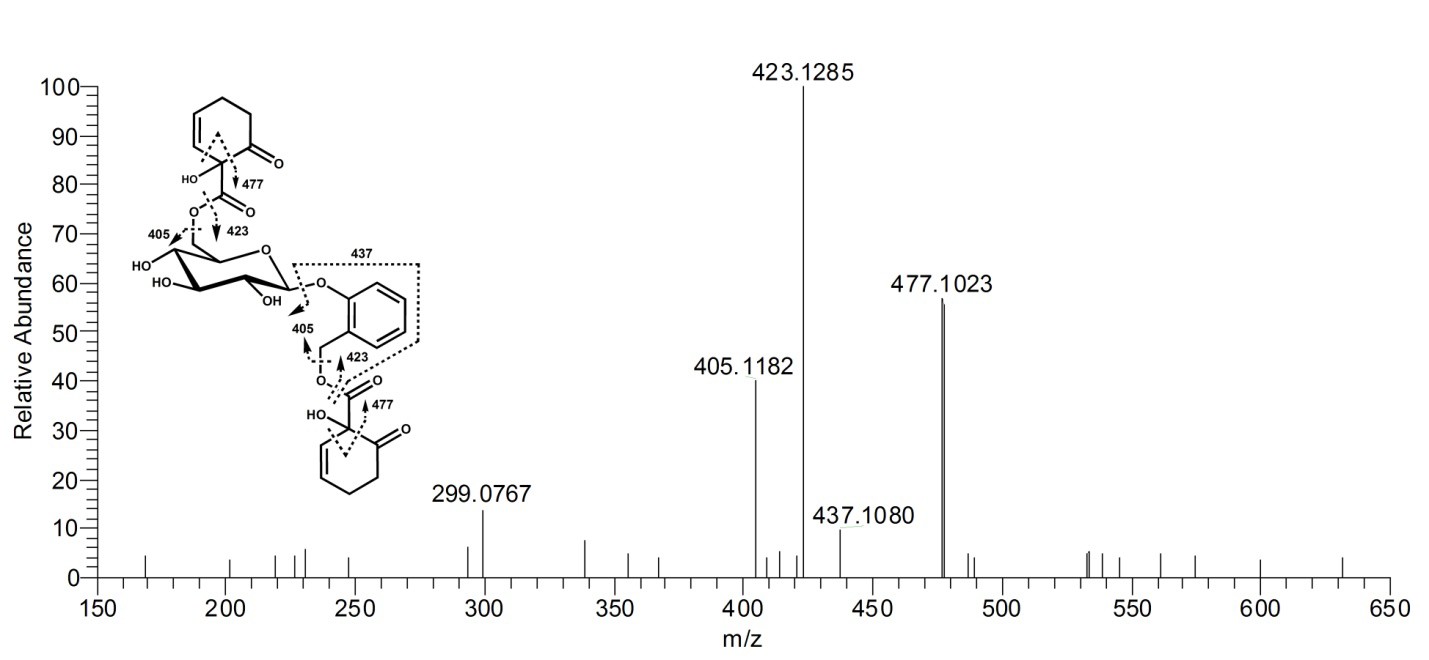


**HCH-tremulacin** - Used [M-H]- *m*/*z* 665.186 for fragmentation


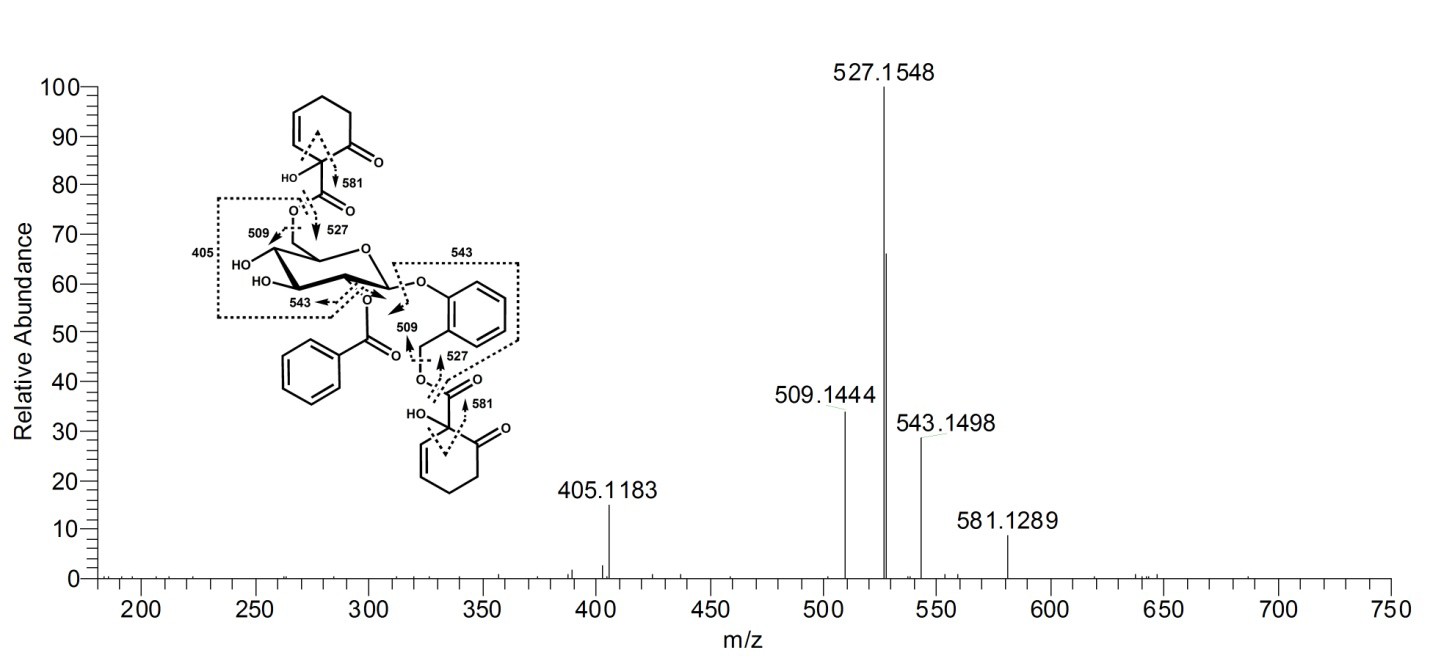


**Cinnamoylsalicin Isomer 2** (the putative 2’-(*E*)-cinnamoylsalicin isomer shown). Used [M- H+FA]- *m*/*z* 461.145 for fragmentation


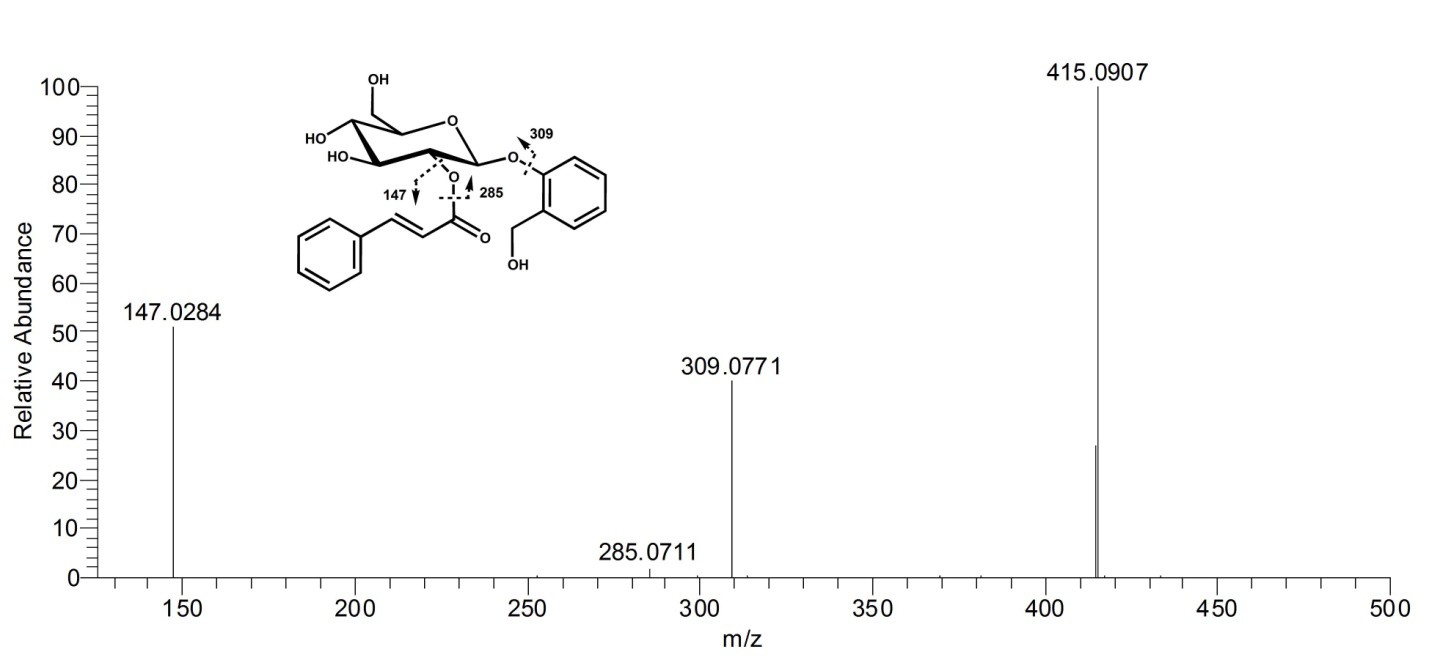


**Acetylcinnamoylsalicortin Isomer 2** (the putative 6’-acetyl-2’-(*E*)-cinnamoylsalicortin isomer shown) - Used [M-H]- *m*/*z* 595.179 for fragmentation


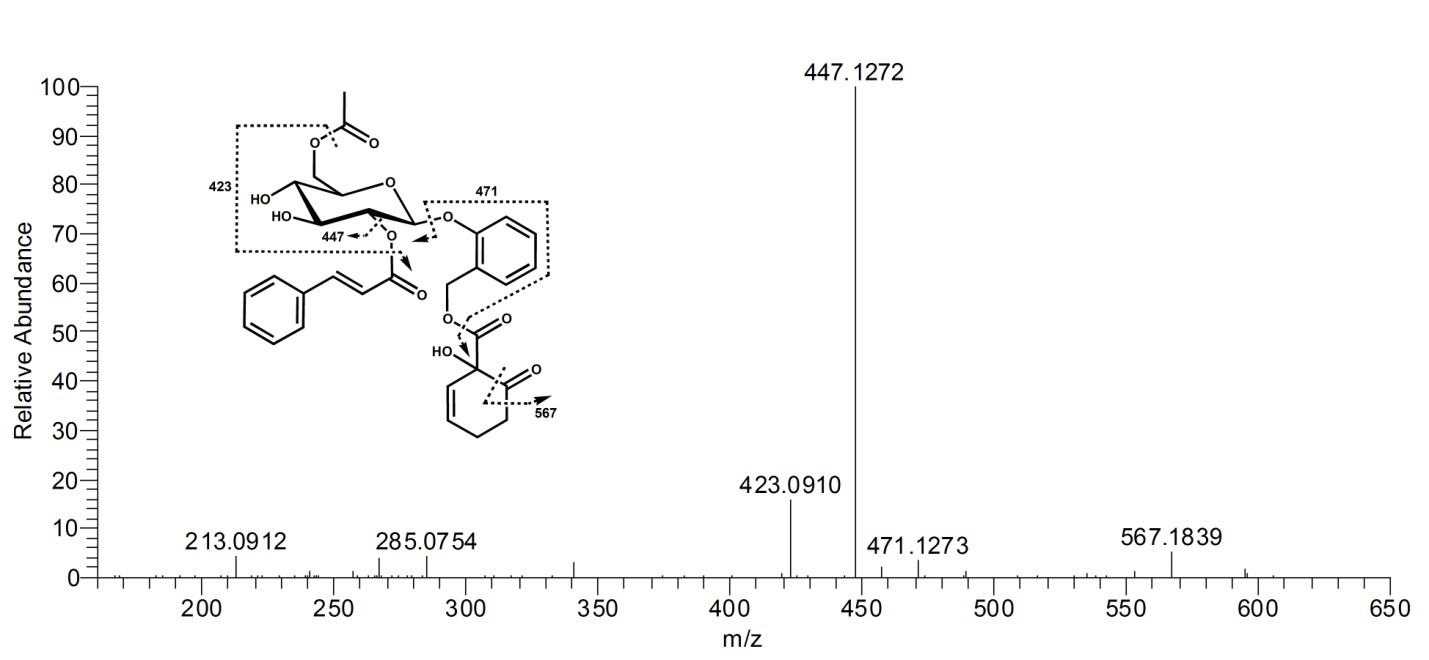


**HCH-cinnamoylsalicortin Isomer 2** (the putative 6’-HCH-2’-(*E*)-cinnamoylsalicortin isomer shown) - Used [M-H]- *m*/*z* 691.202 for fragmentation


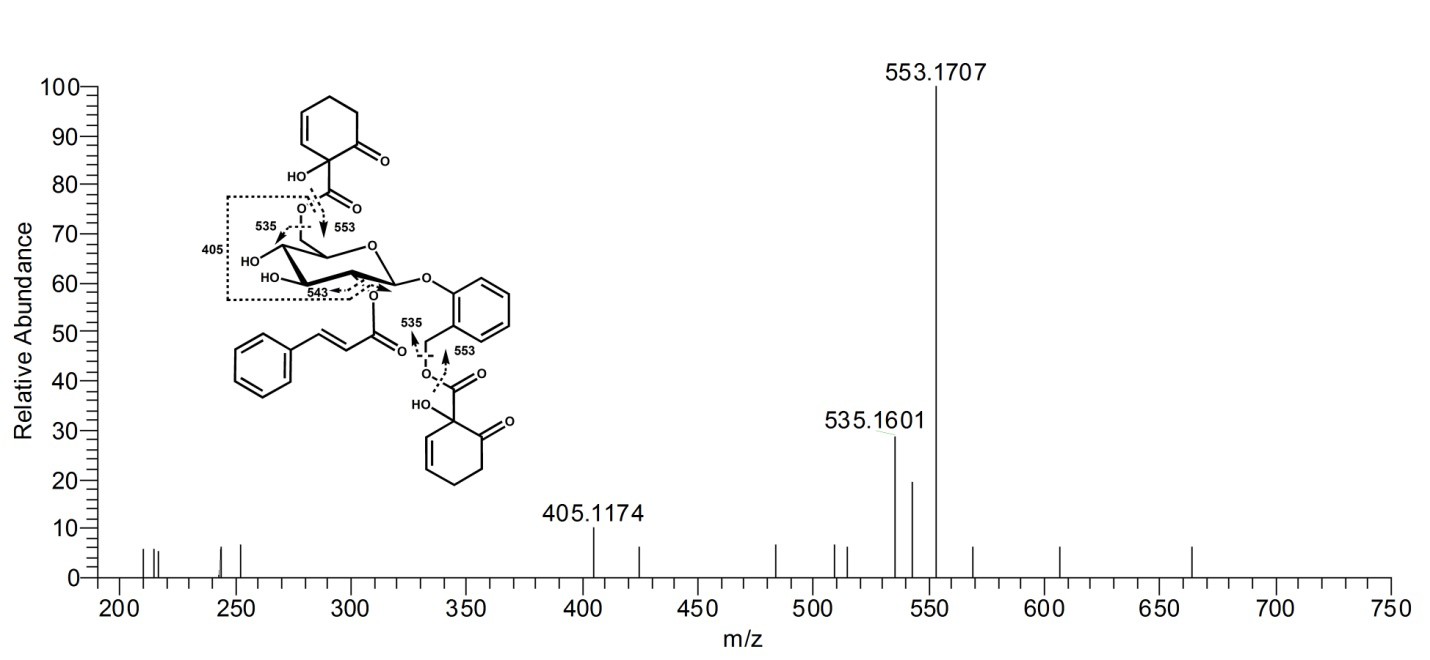


**Lasiandrin (HCH-2’-acetylsalicortin)** - Used [M-H]- *m*/*z* 603.172 for fragmentation


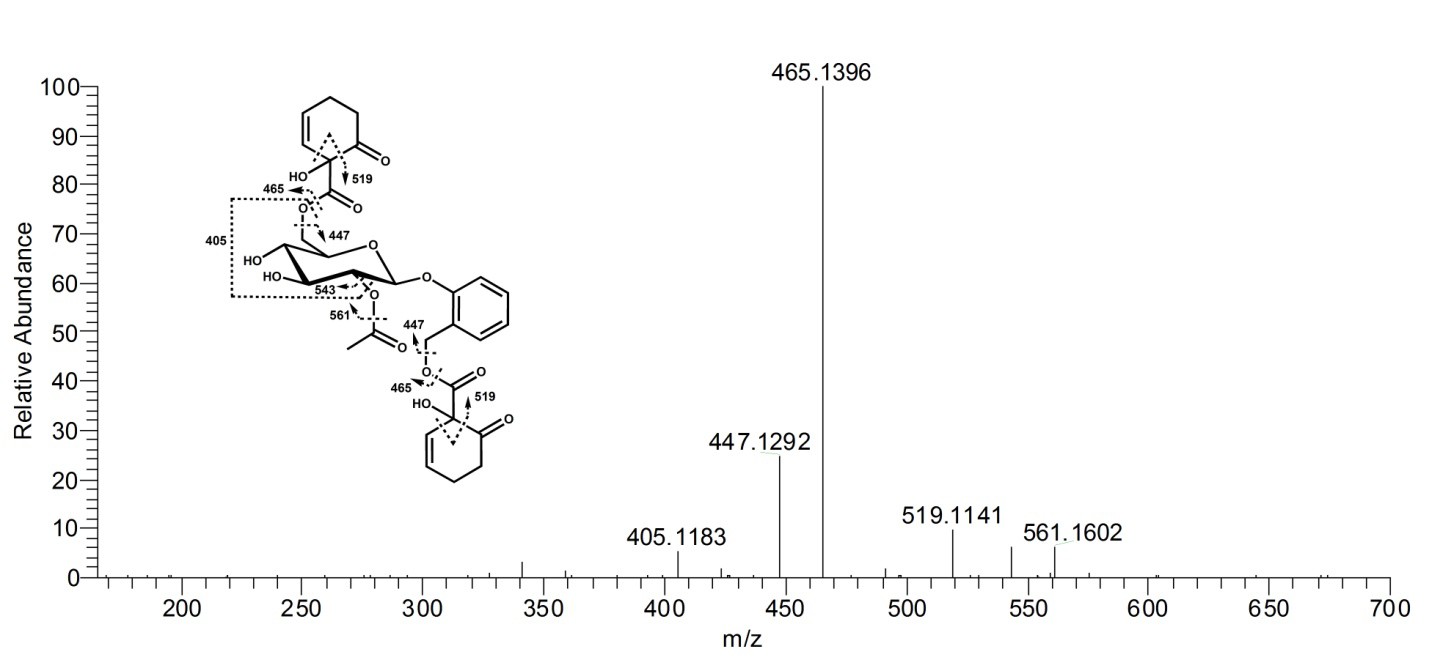

Supplement: Material S1 — Salicinoid identification: Exact masses of 55 salicinoid compounds (Table S1), Literature references (List S1), UV spectra of nine new salicinoids found in the P. tremula foliage (Fig. S1 a), and high-resolution MS/MS (Fig. S1 b). (DOCX) [file pone.0107189.s001.docx]
