# Supplementary material for: No Evidence of Geographical Structure of Salicinoid Chemotypes within Populus Tremula
Source: PLoS One. 2014 Oct 9;9(10):e107189. doi: 10.1371/journal.pone.0107189 (PMC4191948; doi:10.1371/journal.pone.0107189)
Supplement: Material S2 — Average percentages of 19 salicinoids from the foliage of different Populus tremula clones (Clone), grown in two different environments (Evir: GH = greenhouse, Sävar = field) and mg g−1 for field trees. Chemo = chemotype: CN = 2′-cinnamoyl, AC = 2′- acetyl, CN-AC = 2′-cinnamoyl/2′-acetyl, and TL = tremuloides-like. Salicinoids: 1 = salicortin, 2 = tremulacin, 3 = salicin, 4 = tremuloidin, 5 = HCH-salicortin, 6 = HCH-tremulacin, 7 = salicyloylsalicin, 8 = 6′-acetyl-tremulacin, 9 = 2′-(Z)-cinnamoylsalicortin, 10 = 2′-(E)- cinnamoylsalicortin, 11 = cinnamoylsalicin I1, 12 = cinnamoylsalicin I2, 13 = acetylcinnamoylsalicortin I1, 14 = acetylcinnamoylsalicortin I2, 15 = HCH-cinnamoylsalicortin I1, 16 = HCH-cinnamoylsalicortin I2, 17 = 2′-acetylsalicortin, 18 = 2′-acetylsalicin, 19 = lasiandrin (HCH-2'-acetylsalicortin). I1 and I2 = isomers 1 and 2, respectively. (DOCX) [file pone.0107189.s002.docx]

**Table S2.** Average percentages of 19 salicinoids from the foliage of different *Populus tremula* clones (Clone), grown in two different environments (Evir: GH = greenhouse, Sävar = field) and mg g-1 for field trees. Chemo = chemotype: CN = 2’-cinnamoyl, AC = 2’- acetyl, CN-AC = 2’-cinnamoyl/2’-acetyl, and TL = *tremuloides*-like. Salicinoids: 1 = salicortin, 2 = tremulacin, 3 = salicin, 4 = tremuloidin, 5

= HCH-salicortin, 6 = HCH-tremulacin, 7 = salicyloylsalicin, 8 = 6’-acetyl-tremulacin, 9 = 2’-(*Z*)-cinnamoylsalicortin, 10 = 2’-(*E*)- cinnamoylsalicortin, 11 = cinnamoylsalicin I*1*, 12 = cinnamoylsalicin I*2*, 13 = acetylcinnamoylsalicortin I*1*, 14 = acetylcinnamoylsalicortin I*2*, 15 = HCH-cinnamoylsalicortin I*1*, 16 = HCH-cinnamoylsalicortin I*2*, 17 = 2’-acetylsalicortin, 18 = 2’-acetylsalicin, 19 = lasiandrin

(HCH-2'-acetylsalicortin). I*1* and I*2* = isomers 1 and 2, respectively

**Clone Chemo Envir**

**Salicinoids**

**1** CN GH

| **1** | **2** | **3** | **4** | **5** | **6** | **7** | **8** | **9** | **10** | **11** | **12** | **13** | **14** | **15** | **16** | **17** | **18** | **19** | **mg g-1** |
| --- | --- | --- | --- | --- | --- | --- | --- | --- | --- | --- | --- | --- | --- | --- | --- | --- | --- | --- | --- |
| 40.3 | 11.1 | 6.0 | 2.8 | 0.9 | 0.1 | 0.2 | 0.6 | 12.9 | 13.7 | 0.6 | 2.8 | 0.5 | 4.5 | 0.0 | 0.1 | 2.8 | 0.1 | 0.0 | --- |
| 44.1 | 35.7 | 5.8 | 7.2 | 1.2 | 0.7 | 0.1 | 4.6 | 0.0 | 0.1 | 0.0 | 0.0 | 0.0 | 0.0 | 0.0 | 0.0 | 0.4 | 0.0 | 0.0 | --- |
| 48.8 | 37.7 | 3.9 | 5.2 | 1.9 | 1.0 | 0.1 | 0.0 | 0.0 | 0.1 | 0.0 | 0.0 | 0.0 | 0.0 | 0.0 | 0.0 | 1.0 | 0.1 | 0.2 | --- |
| 47.8 | 11.8 | 5.2 | 0.9 | 2.3 | 0.1 | 0.2 | 0.0 | 12.0 | 14.8 | 0.3 | 2.2 | 0.0 | 0.0 | 0.0 | 0.1 | 2.2 | 0.1 | 0.1 | --- |
| 48.2 | 34.4 | 5.5 | 6.0 | 2.1 | 1.4 | 0.1 | 0.0 | 0.0 | 0.1 | 0.0 | 0.0 | 0.0 | 0.0 | 0.0 | 0.0 | 2.2 | 0.1 | 0.1 | --- |
| 43.2 | 34.3 | 6.0 | 7.1 | 1.9 | 0.8 | 0.1 | 4.5 | 0.0 | 0.1 | 0.0 | 0.0 | 0.0 | 0.0 | 0.0 | 0.0 | 1.7 | 0.1 | 0.2 | --- |
| 37.3 | 11.4 | 6.2 | 3.8 | 0.6 | 0.0 | 0.3 | 0.0 | 15.5 | 16.4 | 0.6 | 3.1 | 0.0 | 0.0 | 0.0 | 0.1 | 3.6 | 1.0 | 0.1 | --- |
| 47.9 | 39.4 | 3.3 | 5.8 | 1.2 | 0.6 | 0.2 | 0.1 | 0.0 | 0.1 | 0.0 | 0.0 | 0.0 | 0.0 | 0.0 | 0.0 | 0.8 | 0.3 | 0.1 | --- |
| 43.7 | 36.9 | 4.8 | 4.1 | 2.6 | 1.1 | 0.1 | 4.9 | 0.0 | 0.1 | 0.0 | 0.3 | 0.0 | 0.0 | 0.0 | 0.0 | 1.2 | 0.0 | 0.1 | --- |
| 38.0 | 9.4 | 4.2 | 0.9 | 1.0 | 0.0 | 0.1 | 0.8 | 16.4 | 13.3 | 0.9 | 2.4 | 2.2 | 4.5 | 0.0 | 0.0 | 5.5 | 0.3 | 0.1 | --- |
| 38.5 | 9.7 | 8.4 | 2.8 | 0.9 | 0.1 | 0.1 | 0.3 | 13.9 | 15.4 | 1.2 | 1.9 | 0.5 | 2.4 | 0.0 | 0.1 | 3.1 | 0.6 | 0.1 | --- |
| 41.2 | 11.4 | 5.4 | 1.1 | 2.7 | 0.2 | 0.4 | 0.5 | 12.0 | 14.7 | 0.2 | 2.5 | 0.3 | 3.8 | 0.0 | 0.2 | 2.9 | 0.1 | 0.1 | --- |
| 44.3 | 17.3 | 5.1 | 2.7 | 0.5 | 0.1 | 0.1 | 0.0 | 12.9 | 14.6 | 0.3 | 1.5 | 0.0 | 0.0 | 0.0 | 0.0 | 0.5 | 0.0 | 0.0 | --- |
| 44.0 | 13.6 | 0.0 | 0.8 | 1.2 | 0.1 | 0.1 | 0.3 | 15.1 | 16.3 | 0.3 | 2.1 | 0.2 | 2.9 | 0.0 | 0.1 | 2.6 | 0.1 | 0.1 | --- |
| 37.6 | 38.3 | 7.1 | 8.5 | 0.5 | 0.2 | 0.2 | 5.2 | 0.0 | 0.1 | 0.0 | 0.0 | 0.0 | 0.0 | 0.0 | 0.0 | 2.1 | 0.2 | 0.0 | --- |
| 37.7 | 11.0 | 6.0 | 2.3 | 1.4 | 0.1 | 0.1 | 0.9 | 13.7 | 14.7 | 0.4 | 2.0 | 1.0 | 5.0 | 0.0 | 0.1 | 3.1 | 0.2 | 0.1 | --- |
| 39.2 | 36.0 | 9.2 | 11.4 | 0.3 | 0.2 | 0.1 | 2.3 | 0.0 | 0.1 | 0.0 | 0.0 | 0.0 | 0.0 | 0.0 | 0.0 | 0.9 | 0.1 | 0.0 | --- |
| 44.5 | 12.3 | 3.2 | 1.4 | 4.8 | 0.5 | 0.1 | 0.1 | 14.8 | 14.0 | 0.8 | 1.2 | 0.1 | 0.6 | 0.1 | 0.1 | 1.2 | 0.1 | 0.0 | --- |
| 42.1 | 11.5 | 6.9 | 2.6 | 0.3 | 0.0 | 0.1 | 0.0 | 16.7 | 15.1 | 0.9 | 1.9 | 0.0 | 0.0 | 0.0 | 0.0 | 1.6 | 0.1 | 0.1 | --- |
| 41.6 | 10.8 | 4.8 | 1.0 | 0.7 | 0.0 | 0.2 | 0.1 | 17.3 | 18.1 | 0.2 | 2.4 | 0.0 | 0.1 | 0.0 | 0.1 | 2.5 | 0.1 | 0.0 | --- |
| 47.0 | 36.4 | 5.4 | 6.3 | 1.2 | 0.5 | 0.1 | 2.1 | 0.0 | 0.1 | 0.0 | 0.0 | 0.0 | 0.0 | 0.0 | 0.0 | 0.8 | 0.0 | 0.0 | --- |
| 37.8 | 10.5 | 5.7 | 2.1 | 2.5 | 0.1 | 0.2 | 0.0 | 15.5 | 17.5 | 0.3 | 3.1 | 0.0 | 0.0 | 0.0 | 0.4 | 3.3 | 0.6 | 0.1 | --- |
| 46.3 | 32.0 | 6.5 | 7.6 | 2.1 | 0.9 | 0.1 | 3.8 | 0.0 | 0.1 | 0.0 | 0.0 | 0.0 | 0.0 | 0.0 | 0.0 | 0.5 | 0.0 | 0.1 | --- |
| 32.1 | 11.0 | 9.9 | 3.9 | 0.3 | 0.1 | 0.1 | 0.8 | 10.1 | 15.9 | 0.5 | 2.8 | 0.2 | 4.2 | 0.0 | 0.1 | 6.0 | 1.8 | 0.2 | --- |

**3** TL GH

**4** TL GH

**5** CN GH

**6** TL GH

**7** TL GH

**9** CN GH

**10** TL GH

**11** TL GH

**12** CN GH

**13** CN GH

**14** CN GH

**15** CN GH

**16** CN GH

**18** TL GH

**19** CN GH

**20** TL GH

**21** CN GH

**22** CN GH

**23** CN GH

**24** TL GH

**25** CN GH

**26** TL GH

**28** CN GH

| **30** | CN | GH | 42.3 | 11.1 | 3.9 | 0.9 | 2.5 | 0.2 | 0.1 | 0.8 | 15.0 | 13.2 | 0.2 | 2.0 | 1.0 | 4.0 | 0.0 | 0.0 | 2.6 | 0.0 | 0.1 | --- |
| --- | --- | --- | --- | --- | --- | --- | --- | --- | --- | --- | --- | --- | --- | --- | --- | --- | --- | --- | --- | --- | --- | --- |
| **31** | CN | GH | 36.1 | 14.0 | 7.1 | 4.2 | 0.9 | 0.2 | 0.2 | 1.0 | 12.0 | 14.3 | 0.6 | 2.2 | 0.4 | 3.4 | 0.0 | 0.0 | 2.9 | 0.2 | 0.2 | --- |
| **32** | CN | GH | 37.9 | 9.2 | 4.4 | 1.3 | 1.3 | 0.1 | 0.2 | 1.2 | 13.8 | 14.6 | 0.3 | 2.6 | 1.3 | 6.5 | 0.0 | 0.1 | 5.2 | 0.2 | 0.1 | --- |
| **33** | CN | GH | 39.7 | 8.9 | 8.9 | 3.0 | 4.5 | 0.5 | 0.1 | 0.3 | 12.0 | 13.2 | 2.0 | 2.6 | 0.3 | 1.8 | 0.1 | 0.4 | 1.5 | 0.2 | 0.1 | --- |
| **34** | CN | GH | 43.6 | 10.0 | 6.6 | 2.2 | 2.3 | 0.2 | 0.1 | 0.8 | 9.9 | 13.2 | 0.5 | 2.5 | 0.6 | 3.9 | 0.0 | 0.1 | 3.0 | 0.2 | 0.2 | --- |
| **35** | CN | GH | 41.9 | 17.1 | 5.4 | 3.6 | 0.3 | 0.1 | 0.1 | 0.9 | 9.0 | 10.5 | 0.3 | 1.5 | 0.8 | 4.5 | 0.0 | 0.0 | 3.9 | 0.1 | 0.0 | --- |
| **36** | CN | GH | 33.7 | 10.5 | 4.8 | 1.4 | 1.5 | 0.1 | 0.1 | 1.2 | 14.6 | 14.8 | 0.3 | 2.4 | 1.4 | 6.8 | 0.0 | 0.1 | 5.8 | 0.3 | 0.1 | --- |
| **37** | TL | GH | 45.1 | 35.0 | 5.4 | 6.6 | 0.6 | 0.2 | 0.1 | 5.7 | 0.0 | 0.1 | 0.0 | 0.3 | 0.0 | 0.0 | 0.0 | 0.0 | 0.9 | 0.0 | 0.0 | --- |
| **39** | CN | GH | 39.3 | 7.8 | 4.9 | 0.6 | 1.5 | 0.1 | 0.1 | 0.6 | 12.9 | 15.7 | 0.2 | 2.5 | 0.7 | 6.0 | 0.0 | 0.1 | 6.4 | 0.1 | 0.4 | --- |
| **40** | CN | GH | 37.7 | 12.5 | 4.6 | 3.2 | 0.3 | 0.0 | 0.1 | 0.8 | 12.6 | 14.7 | 0.3 | 2.2 | 0.3 | 3.5 | 0.0 | 0.0 | 6.4 | 0.7 | 0.1 | --- |
| **42** | CN | GH | 46.1 | 14.4 | 4.5 | 1.2 | 1.2 | 0.1 | 0.1 | 0.4 | 11.3 | 15.2 | 0.2 | 1.4 | 0.2 | 1.8 | 0.0 | 0.1 | 1.9 | 0.0 | 0.0 | --- |
| **43** | CN | GH | 43.2 | 12.1 | 9.6 | 3.6 | 1.3 | 0.1 | 0.1 | 0.0 | 7.8 | 16.0 | 0.3 | 2.2 | 0.0 | 0.1 | 0.0 | 0.0 | 3.0 | 0.4 | 0.2 | --- |
| **44** | CN | GH | 37.8 | 12.3 | 6.7 | 2.5 | 3.4 | 0.5 | 0.2 | 0.5 | 13.1 | 14.5 | 0.6 | 2.1 | 0.4 | 2.8 | 0.1 | 0.2 | 1.9 | 0.2 | 0.1 | --- |
| **45** | TL | GH | 45.2 | 34.7 | 6.2 | 8.4 | 0.4 | 0.1 | 0.1 | 4.6 | 0.0 | 0.1 | 0.0 | 0.0 | 0.0 | 0.0 | 0.0 | 0.0 | 0.3 | 0.0 | 0.0 | --- |
| **46** | CN | GH | 40.8 | 13.3 | 5.2 | 5.2 | 0.4 | 0.1 | 0.1 | 0.8 | 8.4 | 16.6 | 0.5 | 2.2 | 0.3 | 4.2 | 0.0 | 0.0 | 1.8 | 0.1 | 0.1 | --- |
| **47** | AC | GH | 33.4 | 25.6 | 3.3 | 3.3 | 0.2 | 0.1 | 0.1 | 2.2 | 0.1 | 0.3 | 0.0 | 0.0 | 0.0 | 0.0 | 0.0 | 0.0 | 28.3 | 3.1 | 0.1 | --- |
| **48** | CN | GH | 42.0 | 14.1 | 6.4 | 3.0 | 0.5 | 0.1 | 0.1 | 0.0 | 12.4 | 17.7 | 0.3 | 2.1 | 0.0 | 0.1 | 0.0 | 0.0 | 0.9 | 0.1 | 0.0 | --- |
| **49** | CN | GH | 44.7 | 12.7 | 5.5 | 1.5 | 0.5 | 0.1 | 0.2 | 0.0 | 14.6 | 16.2 | 0.3 | 2.0 | 0.0 | 0.1 | 0.0 | 0.0 | 1.4 | 0.1 | 0.1 | --- |
| **50** | CN | GH | 36.3 | 10.4 | 5.5 | 2.7 | 0.7 | 0.1 | 0.2 | 1.0 | 14.9 | 15.2 | 0.7 | 2.7 | 0.8 | 5.7 | 0.0 | 0.0 | 3.1 | 0.1 | 0.0 | --- |
| **52** | CN | GH | 39.6 | 12.7 | 6.6 | 2.9 | 1.0 | 0.1 | 0.1 | 0.1 | 10.3 | 17.1 | 0.1 | 2.0 | 0.0 | 0.2 | 0.0 | 0.1 | 5.9 | 0.9 | 0.2 | --- |
| **53** | TL | GH | 42.7 | 35.6 | 6.7 | 9.1 | 0.4 | 0.1 | 0.1 | 4.8 | 0.0 | 0.1 | 0.0 | 0.0 | 0.0 | 0.0 | 0.0 | 0.0 | 0.3 | 0.0 | 0.0 | --- |
| **54** | TL | GH | 42.9 | 34.9 | 5.5 | 6.3 | 0.9 | 0.3 | 0.1 | 7.9 | 0.0 | 0.1 | 0.0 | 0.0 | 0.0 | 0.0 | 0.0 | 0.0 | 1.2 | 0.0 | 0.0 | --- |
| **55** | CN-AC | GH | 24.8 | 8.0 | 5.2 | 2.4 | 0.1 | 0.0 | 0.1 | 0.4 | 10.8 | 12.6 | 1.2 | 2.0 | 0.8 | 2.6 | 0.0 | 0.0 | 21.7 | 7.0 | 0.3 | --- |
| **56** | AC | GH | 32.3 | 29.7 | 3.3 | 4.0 | 0.7 | 0.3 | 0.1 | 0.1 | 0.0 | 0.3 | 0.0 | 0.0 | 0.0 | 0.0 | 0.0 | 0.0 | 25.7 | 3.0 | 0.5 | --- |
| **57** | CN | GH | 43.3 | 9.7 | 4.3 | 1.0 | 2.7 | 0.1 | 0.1 | 0.6 | 14.7 | 13.9 | 0.3 | 1.7 | 0.8 | 4.0 | 0.0 | 0.1 | 2.5 | 0.0 | 0.0 | --- |
| **59** | CN | GH | 35.7 | 11.9 | 4.1 | 1.5 | 1.5 | 0.3 | 0.1 | 1.8 | 13.7 | 15.2 | 0.2 | 2.1 | 1.3 | 6.1 | 0.0 | 0.1 | 4.0 | 0.1 | 0.1 | --- |
| **60** | AC | GH | 29.9 | 25.2 | 3.7 | 3.9 | 0.5 | 0.1 | 0.1 | 5.6 | 0.1 | 0.2 | 0.0 | 0.0 | 0.0 | 0.0 | 0.0 | 0.0 | 26.4 | 3.8 | 0.6 | --- |
| **61** | AC | GH | 32.0 | 24.8 | 2.7 | 4.6 | 0.5 | 0.1 | 0.1 | 3.1 | 0.0 | 0.1 | 0.0 | 0.0 | 0.0 | 0.0 | 0.0 | 0.0 | 26.3 | 5.0 | 0.6 | --- |
| **62** | TL | GH | 42.5 | 37.5 | 6.1 | 7.1 | 1.4 | 1.3 | 0.1 | 0.0 | 0.0 | 0.2 | 0.0 | 0.0 | 0.0 | 0.0 | 0.0 | 0.0 | 3.1 | 0.6 | 0.1 | --- |
| **64** | CN | GH | 44.4 | 14.2 | 4.8 | 1.3 | 0.8 | 0.1 | 0.2 | 0.8 | 11.2 | 14.0 | 0.3 | 1.5 | 0.3 | 2.4 | 0.0 | 0.0 | 3.6 | 0.1 | 0.1 | --- |
| **66** | CN | GH | 35.9 | 12.0 | 6.2 | 4.7 | 0.3 | 0.1 | 0.1 | 1.4 | 12.5 | 14.3 | 0.7 | 2.6 | 0.8 | 5.3 | 0.0 | 0.0 | 2.8 | 0.3 | 0.0 | --- |
| **67** | AC | GH | 29.1 | 28.3 | 3.2 | 4.3 | 0.5 | 0.2 | 0.1 | 4.8 | 0.1 | 0.1 | 0.0 | 0.0 | 0.0 | 0.0 | 0.0 | 0.0 | 25.7 | 3.3 | 0.3 | --- |
| **68** | CN | GH | 40.9 | 13.5 | 5.7 | 2.7 | 2.5 | 0.4 | 0.2 | 0.0 | 14.2 | 15.0 | 0.4 | 2.3 | 0.0 | 0.0 | 0.0 | 0.1 | 1.6 | 0.2 | 0.0 | --- |
| **69** | TL | GH | 47.0 | 37.0 | 5.7 | 6.0 | 2.3 | 1.6 | 0.1 | 0.0 | 0.0 | 0.1 | 0.0 | 0.0 | 0.0 | 0.0 | 0.0 | 0.0 | 0.1 | 0.0 | 0.0 | --- |
| **70** | TL | GH | 43.9 | 34.3 | 7.6 | 10.1 | 0.3 | 0.1 | 0.1 | 2.7 | 0.0 | 0.1 | 0.0 | 0.0 | 0.0 | 0.0 | 0.0 | 0.0 | 0.7 | 0.1 | 0.0 | --- |
| **71** | TL | GH | 47.6 | 33.1 | 6.2 | 6.2 | 1.1 | 0.5 | 0.1 | 4.8 | 0.0 | 0.1 | 0.0 | 0.0 | 0.0 | 0.0 | 0.0 | 0.0 | 0.4 | 0.0 | 0.0 | --- |
| **72** | TL | GH | 44.6 | 32.5 | 6.3 | 5.9 | 4.4 | 2.5 | 0.1 | 2.1 | 0.0 | 0.1 | 0.0 | 0.0 | 0.0 | 0.0 | 0.0 | 0.0 | 1.2 | 0.1 | 0.2 | --- |
| **73** | TL | GH | 54.3 | 34.0 | 0.0 | 5.0 | 1.0 | 0.3 | 0.1 | 4.1 | 0.0 | 0.3 | 0.0 | 0.0 | 0.0 | 0.0 | 0.0 | 0.0 | 0.8 | 0.0 | 0.0 | --- |
| **74** | TL | GH | 50.8 | 30.6 | 7.0 | 4.9 | 2.9 | 0.8 | 0.1 | 1.5 | 0.0 | 0.1 | 0.0 | 0.0 | 0.0 | 0.0 | 0.0 | 0.0 | 1.2 | 0.2 | 0.1 | --- |

| **77** | TL | GH | 43.0 | 34.5 | 4.9 | 9.5 | 1.0 | 0.6 | 0.2 | 5.4 | 0.1 | 0.2 | 0.0 | 0.0 | 0.0 | 0.0 | 0.0 | 0.0 | 0.5 | 0.0 | 0.0 | --- |
| --- | --- | --- | --- | --- | --- | --- | --- | --- | --- | --- | --- | --- | --- | --- | --- | --- | --- | --- | --- | --- | --- | --- |
| **78** | TL | GH | 52.5 | 30.7 | 5.6 | 5.0 | 1.0 | 0.2 | 0.1 | 3.7 | 0.0 | 0.0 | 0.0 | 0.0 | 0.0 | 0.0 | 0.0 | 0.0 | 1.2 | 0.0 | 0.1 | --- |
| **79** | TL | GH | 48.1 | 31.1 | 6.5 | 8.1 | 2.2 | 0.9 | 0.1 | 0.6 | 0.0 | 0.1 | 0.0 | 0.0 | 0.0 | 0.0 | 0.0 | 0.0 | 2.0 | 0.3 | 0.2 | --- |
| **80** | CN | GH | 42.1 | 11.4 | 3.2 | 3.5 | 1.2 | 0.2 | 0.1 | 0.7 | 13.8 | 13.4 | 1.8 | 1.6 | 1.0 | 2.7 | 0.0 | 0.0 | 2.8 | 0.3 | 0.1 | --- |
| **81** | TL | GH | 42.6 | 37.4 | 5.7 | 5.7 | 1.0 | 0.3 | 0.1 | 4.2 | 0.0 | 0.1 | 0.0 | 0.0 | 0.0 | 0.0 | 0.0 | 0.0 | 2.7 | 0.1 | 0.1 | --- |
| **82** | CN | GH | 36.5 | 16.2 | 4.7 | 2.1 | 5.2 | 1.7 | 0.2 | 1.2 | 12.2 | 11.9 | 0.2 | 1.6 | 0.4 | 3.1 | 0.1 | 0.4 | 2.1 | 0.1 | 0.1 | --- |
| **84** | CN | GH | 45.1 | 11.7 | 5.1 | 0.9 | 1.8 | 0.2 | 0.1 | 0.6 | 12.9 | 14.4 | 0.2 | 1.6 | 0.5 | 2.6 | 0.0 | 0.0 | 2.2 | 0.0 | 0.1 | --- |
| **85** | TL | GH | 38.6 | 38.4 | 6.0 | 7.9 | 0.9 | 0.4 | 0.1 | 6.3 | 0.0 | 0.1 | 0.0 | 0.0 | 0.0 | 0.0 | 0.0 | 0.0 | 1.0 | 0.1 | 0.1 | --- |
| **86** | AC | GH | 27.7 | 27.8 | 4.0 | 5.6 | 0.4 | 0.1 | 0.1 | 0.0 | 0.0 | 0.2 | 0.0 | 0.0 | 0.0 | 0.0 | 0.0 | 0.0 | 27.1 | 6.6 | 0.2 | --- |
| **87** | TL | GH | 39.4 | 39.6 | 5.6 | 5.5 | 1.9 | 0.9 | 0.1 | 4.4 | 0.0 | 0.1 | 0.0 | 0.0 | 0.0 | 0.0 | 0.0 | 0.0 | 2.2 | 0.1 | 0.1 | --- |
| **88** | CN | GH | 39.6 | 11.8 | 6.4 | 2.8 | 1.5 | 0.2 | 0.2 | 1.2 | 13.0 | 13.2 | 0.8 | 2.0 | 1.0 | 3.9 | 0.0 | 0.1 | 2.2 | 0.1 | 0.0 | --- |
| **89** | AC | GH | 28.3 | 26.4 | 3.6 | 5.4 | 0.2 | 0.0 | 0.0 | 4.4 | 0.0 | 0.1 | 0.1 | 0.1 | 0.0 | 0.0 | 0.0 | 0.0 | 26.5 | 4.8 | 0.1 | --- |
| **90** | TL | GH | 48.0 | 31.9 | 6.4 | 5.4 | 2.1 | 0.8 | 0.1 | 4.4 | 0.0 | 0.0 | 0.0 | 0.0 | 0.0 | 0.0 | 0.0 | 0.0 | 0.7 | 0.0 | 0.1 | --- |
| **91** | TL | GH | 43.1 | 37.2 | 5.1 | 6.4 | 0.7 | 0.2 | 0.1 | 6.0 | 0.0 | 0.1 | 0.0 | 0.0 | 0.0 | 0.0 | 0.0 | 0.0 | 1.1 | 0.0 | 0.0 | --- |
| **92** | TL | GH | 46.8 | 39.2 | 5.6 | 6.6 | 0.8 | 0.3 | 0.1 | 0.0 | 0.0 | 0.3 | 0.0 | 0.0 | 0.0 | 0.0 | 0.0 | 0.0 | 0.2 | 0.0 | 0.0 | --- |
| **93** | CN | GH | 41.4 | 10.7 | 0.0 | 1.2 | 0.5 | 0.1 | 0.2 | 0.7 | 14.1 | 17.0 | 0.3 | 2.6 | 0.7 | 5.8 | 0.0 | 0.0 | 4.5 | 0.2 | 0.0 | --- |
| **95** | CN | GH | 43.2 | 14.5 | 7.1 | 3.2 | 2.7 | 0.6 | 0.1 | 0.1 | 12.0 | 13.2 | 0.4 | 1.3 | 0.0 | 0.5 | 0.0 | 0.1 | 1.0 | 0.0 | 0.0 | --- |
| **96** | TL | GH | 48.1 | 33.7 | 6.3 | 5.1 | 2.4 | 1.0 | 0.2 | 1.8 | 0.1 | 0.4 | 0.0 | 0.0 | 0.0 | 0.0 | 0.0 | 0.0 | 0.8 | 0.1 | 0.1 | --- |
| **97** | CN | GH | 41.5 | 15.3 | 5.1 | 2.8 | 2.5 | 0.5 | 0.1 | 0.0 | 13.3 | 15.4 | 0.2 | 1.4 | 0.0 | 0.0 | 0.0 | 0.1 | 1.4 | 0.1 | 0.0 | --- |
| **98** | TL | GH | 43.9 | 34.1 | 6.9 | 6.8 | 2.8 | 1.7 | 0.1 | 3.4 | 0.0 | 0.1 | 0.0 | 0.0 | 0.0 | 0.0 | 0.0 | 0.0 | 0.2 | 0.0 | 0.0 | --- |
| **99** | CN | GH | 37.1 | 8.6 | 7.5 | 2.2 | 1.4 | 0.1 | 0.1 | 0.4 | 11.8 | 15.7 | 0.8 | 2.4 | 0.3 | 3.7 | 0.0 | 0.0 | 6.3 | 1.1 | 0.5 | --- |
| **100** | TL | GH | 42.8 | 33.3 | 7.4 | 9.1 | 0.4 | 0.1 | 0.1 | 5.0 | 0.0 | 0.1 | 0.0 | 0.0 | 0.0 | 0.0 | 0.0 | 0.0 | 1.7 | 0.1 | 0.0 | --- |
| **102** | CN | GH | 41.6 | 12.5 | 4.7 | 2.2 | 0.2 | 0.0 | 0.2 | 0.9 | 14.0 | 14.7 | 0.2 | 1.9 | 0.5 | 4.1 | 0.0 | 0.0 | 2.2 | 0.1 | 0.0 | --- |
| **103** | TL | GH | 42.3 | 34.2 | 8.2 | 8.7 | 2.0 | 1.4 | 0.1 | 2.7 | 0.0 | 0.1 | 0.0 | 0.0 | 0.0 | 0.0 | 0.0 | 0.0 | 0.4 | 0.0 | 0.0 | --- |
| **104** | CN | GH | 35.4 | 10.8 | 5.8 | 3.1 | 1.5 | 0.2 | 0.2 | 0.7 | 16.0 | 14.4 | 1.2 | 2.3 | 1.6 | 4.4 | 0.0 | 0.1 | 2.0 | 0.2 | 0.0 | --- |
| **105** | CN | GH | 49.1 | 14.9 | 0.0 | 1.4 | 2.2 | 0.3 | 0.1 | 0.6 | 8.2 | 15.8 | 0.2 | 1.5 | 0.2 | 2.0 | 0.0 | 0.0 | 3.5 | 0.0 | 0.0 | --- |
| **106** | TL | GH | 52.9 | 32.6 | 5.8 | 6.6 | 0.9 | 0.1 | 0.1 | 0.0 | 0.1 | 0.6 | 0.0 | 0.0 | 0.0 | 0.0 | 0.0 | 0.0 | 0.1 | 0.0 | 0.0 | --- |
| **109** | CN | GH | 38.7 | 9.7 | 5.6 | 2.8 | 0.3 | 0.0 | 0.1 | 0.5 | 16.4 | 14.5 | 1.0 | 2.3 | 1.4 | 3.7 | 0.0 | 0.0 | 2.8 | 0.3 | 0.0 | --- |
| **110** | TL | GH | 43.8 | 36.1 | 5.5 | 6.2 | 1.3 | 0.6 | 0.1 | 5.6 | 0.1 | 0.1 | 0.0 | 0.0 | 0.0 | 0.0 | 0.0 | 0.0 | 0.7 | 0.0 | 0.0 | --- |
| **112** | CN | GH | 41.8 | 15.8 | 4.4 | 2.9 | 1.4 | 0.2 | 0.6 | 0.1 | 10.0 | 15.8 | 0.1 | 2.0 | 0.0 | 1.7 | 0.0 | 0.0 | 3.0 | 0.2 | 0.1 | --- |
| **113** | CN | GH | 38.7 | 11.7 | 5.7 | 2.3 | 1.6 | 0.1 | 0.2 | 0.6 | 13.9 | 13.9 | 0.5 | 2.4 | 1.0 | 4.5 | 0.0 | 0.1 | 2.6 | 0.1 | 0.0 | --- |
| **114** | CN | GH | 44.0 | 14.5 | 5.8 | 1.8 | 1.5 | 0.2 | 0.1 | 1.3 | 10.7 | 12.3 | 0.3 | 1.4 | 0.6 | 3.4 | 0.0 | 0.0 | 1.7 | 0.0 | 0.0 | --- |
| **115** | AC | GH | 26.8 | 23.8 | 5.0 | 6.1 | 0.2 | 0.0 | 0.0 | 2.8 | 0.0 | 0.5 | 0.0 | 0.0 | 0.0 | 0.0 | 0.0 | 0.0 | 30.6 | 3.7 | 0.3 | --- |
| **116** | CN | GH | 39.1 | 10.8 | 6.5 | 1.8 | 3.7 | 0.3 | 0.1 | 0.9 | 12.2 | 13.8 | 0.3 | 1.7 | 1.1 | 4.3 | 0.1 | 0.2 | 3.0 | 0.2 | 0.1 | --- |
| **1** | CN | Sävar | 38.1 | 21.3 | 7.2 | 1.4 | 2.0 | 0.4 | 0.0 | 0.1 | 11.3 | 16.9 | 0.2 | 0.2 | 0.0 | 0.2 | 0.0 | 0.1 | 0.5 | 0.1 | 0.1 | 58.3 |
| **2** | CN | Sävar | 34.2 | 21.6 | 7.6 | 3.7 | 6.3 | 2.0 | 0.0 | 0.0 | 8.6 | 14.6 | 0.6 | 0.3 | 0.0 | 0.0 | 0.1 | 0.2 | 0.1 | 0.0 | 0.0 | 56.1 |
| **4** | TL | Sävar | 35.0 | 45.6 | 10.0 | 4.7 | 1.8 | 1.4 | 0.0 | 0.0 | 0.0 | 0.1 | 0.0 | 0.0 | 0.0 | 0.0 | 0.0 | 0.0 | 0.6 | 0.6 | 0.2 | 19.1 |
| **5** | CN | Sävar | 42.0 | 18.1 | 4.6 | 0.7 | 4.2 | 0.7 | 0.1 | 0.0 | 11.7 | 16.3 | 0.2 | 0.3 | 0.0 | 0.0 | 0.1 | 0.2 | 0.5 | 0.0 | 0.1 | 46.0 |
| **12** | CN | Sävar | 39.5 | 18.2 | 3.5 | 0.8 | 2.3 | 0.2 | 0.1 | 0.1 | 15.4 | 16.4 | 0.2 | 0.2 | 0.0 | 0.1 | 0.0 | 0.1 | 2.2 | 0.3 | 0.2 | 148.6 |

**14** CN

Sävar

36.9

18.1

5.8

2.1

3.0

0.4

0.1

0.0

14.4

17.1

0.3

0.3

0.0

0.0

0.1

0.1

0.9

0.1

0.1 90.5

| **18** | TL | Sävar | 30.8 | 47.1 | 7.4 | 8.2 | 1.0 | 0.8 | 0.2 | 3.1 | 0.0 | 0.1 | 0.0 | 0.0 | 0.0 | 0.0 | 0.0 | 0.0 | 1.0 | 0.2 | 0.1 | 111.1 |
| --- | --- | --- | --- | --- | --- | --- | --- | --- | --- | --- | --- | --- | --- | --- | --- | --- | --- | --- | --- | --- | --- | --- |
| **22** | CN | Sävar | 35.0 | 18.3 | 5.8 | 1.6 | 0.8 | 0.1 | 0.1 | 0.0 | 16.2 | 19.6 | 0.2 | 0.3 | 0.0 | 0.0 | 0.0 | 0.0 | 1.5 | 0.2 | 0.1 | 232.3 |
| **23** | CN | Sävar | 39.9 | 21.3 | 4.6 | 1.0 | 1.3 | 0.2 | 0.1 | 0.0 | 12.8 | 16.2 | 0.3 | 0.3 | 0.0 | 0.0 | 0.0 | 0.0 | 1.4 | 0.3 | 0.2 | 76.1 |
| **26** | TL | Sävar | 41.3 | 46.1 | 4.0 | 2.1 | 2.8 | 1.5 | 0.1 | 1.9 | 0.0 | 0.1 | 0.0 | 0.0 | 0.0 | 0.0 | 0.0 | 0.0 | 0.1 | 0.0 | 0.0 | 60.9 |
| **28** | CN | Sävar | 26.4 | 18.6 | 9.2 | 5.3 | 1.3 | 0.3 | 0.0 | 0.1 | 13.5 | 17.6 | 1.7 | 0.4 | 0.0 | 0.1 | 0.0 | 0.1 | 2.9 | 2.0 | 0.4 | 54.1 |
| **29** | CN | Sävar | 34.6 | 20.7 | 6.5 | 2.4 | 2.9 | 0.5 | 0.0 | 0.0 | 14.0 | 17.3 | 0.3 | 0.3 | 0.0 | 0.0 | 0.0 | 0.1 | 0.2 | 0.0 | 0.0 | 72.1 |
| **30** | CN | Sävar | 39.1 | 17.2 | 5.7 | 1.1 | 3.4 | 0.9 | 0.1 | 0.2 | 12.6 | 17.3 | 0.2 | 0.3 | 0.1 | 0.6 | 0.1 | 0.1 | 0.6 | 0.1 | 0.1 | 54.4 |
| **34** | CN | Sävar | 37.5 | 20.3 | 8.0 | 2.2 | 4.6 | 0.9 | 0.1 | 0.1 | 9.3 | 13.9 | 0.3 | 0.2 | 0.0 | 0.1 | 0.1 | 0.2 | 1.7 | 0.3 | 0.2 | 31.4 |
| **36** | CN | Sävar | 38.3 | 18.3 | 6.1 | 1.3 | 2.8 | 0.3 | 0.0 | 0.2 | 12.2 | 16.1 | 0.4 | 0.4 | 0.1 | 0.3 | 0.0 | 0.1 | 2.3 | 0.5 | 0.3 | 30.9 |
| **37** | TL | Sävar | 40.8 | 42.6 | 6.4 | 5.6 | 2.1 | 1.2 | 0.1 | 0.8 | 0.0 | 0.1 | 0.0 | 0.0 | 0.0 | 0.0 | 0.0 | 0.0 | 0.3 | 0.0 | 0.0 | 43.9 |
| **40** | CN | Sävar | 36.4 | 24.5 | 6.3 | 2.0 | 1.1 | 0.4 | 0.0 | 0.1 | 9.3 | 11.6 | 0.3 | 0.2 | 0.0 | 0.0 | 0.0 | 0.0 | 5.6 | 1.5 | 0.6 | 28.6 |
| **41** | TL | Sävar | 39.1 | 43.2 | 8.1 | 5.8 | 2.0 | 0.9 | 0.1 | 0.6 | 0.0 | 0.1 | 0.0 | 0.0 | 0.0 | 0.0 | 0.0 | 0.0 | 0.0 | 0.0 | 0.0 | 25.2 |
| **43** | CN | Sävar | 41.2 | 20.8 | 5.7 | 0.9 | 4.6 | 0.8 | 0.0 | 0.0 | 7.2 | 13.3 | 0.2 | 0.2 | 0.0 | 0.0 | 0.1 | 0.1 | 3.6 | 0.6 | 0.7 | 22.5 |
| **47** | AC | Sävar | 21.2 | 28.7 | 3.1 | 0.8 | 0.7 | 0.3 | 0.0 | 0.0 | 0.0 | 0.1 | 0.0 | 0.0 | 0.0 | 0.0 | 0.0 | 0.0 | 40.9 | 2.4 | 1.8 | 21.5 |
| **50** | CN | Sävar | 41.6 | 22.5 | 7.5 | 1.4 | 2.7 | 0.5 | 0.0 | 0.0 | 9.0 | 12.7 | 0.4 | 0.3 | 0.0 | 0.1 | 0.0 | 0.1 | 0.7 | 0.3 | 0.1 | 20.0 |
| **51** | TL | Sävar | 38.0 | 44.3 | 5.2 | 1.6 | 5.3 | 2.8 | 0.0 | 0.2 | 0.1 | 0.1 | 0.0 | 0.0 | 0.0 | 0.0 | 0.0 | 0.0 | 1.5 | 0.3 | 0.6 | 14.5 |
| **53** | TL | Sävar | 35.5 | 41.6 | 11.0 | 9.4 | 1.1 | 0.6 | 0.1 | 0.3 | 0.1 | 0.1 | 0.0 | 0.0 | 0.0 | 0.0 | 0.0 | 0.0 | 0.0 | 0.0 | 0.0 | 26.3 |
| **57** | CN | Sävar | 45.1 | 20.2 | 4.3 | 0.9 | 5.4 | 0.8 | 0.0 | 0.2 | 7.9 | 13.9 | 0.2 | 0.1 | 0.0 | 0.4 | 0.1 | 0.1 | 0.3 | 0.0 | 0.0 | 29.8 |
| **59** | CN | Sävar | 42.4 | 28.6 | 2.8 | 0.5 | 2.3 | 0.6 | 0.1 | 0.5 | 8.8 | 12.2 | 0.1 | 0.1 | 0.0 | 0.2 | 0.0 | 0.0 | 0.5 | 0.0 | 0.1 | 54.6 |
| **60** | AC | Sävar | 31.4 | 32.1 | 2.3 | 1.4 | 0.3 | 0.1 | 0.1 | 3.6 | 0.1 | 0.1 | 0.0 | 0.0 | 0.0 | 0.0 | 0.0 | 0.0 | 26.3 | 1.9 | 0.4 | 99.3 |
| **64** | CN | Sävar | 42.8 | 17.0 | 3.5 | 0.4 | 2.1 | 0.3 | 0.1 | 0.2 | 12.7 | 16.5 | 0.2 | 0.3 | 0.1 | 0.5 | 0.0 | 0.1 | 2.4 | 0.5 | 0.2 | 43.6 |
| **65** | CN-AC | Sävar | 20.2 | 12.1 | 3.8 | 0.4 | 0.7 | 0.2 | 0.0 | 0.0 | 6.6 | 10.5 | 0.2 | 0.2 | 0.0 | 0.0 | 0.0 | 0.0 | 38.7 | 3.8 | 2.5 | 31.3 |
| **67** | AC | Sävar | 22.2 | 31.2 | 4.9 | 4.1 | 0.3 | 0.4 | 0.1 | 1.5 | 0.1 | 0.1 | 0.0 | 0.0 | 0.0 | 0.0 | 0.0 | 0.0 | 27.9 | 6.6 | 0.7 | 79.3 |
| **68** | CN | Sävar | 37.1 | 21.9 | 7.8 | 2.9 | 4.3 | 1.3 | 0.1 | 0.0 | 9.3 | 14.0 | 0.3 | 0.2 | 0.0 | 0.0 | 0.1 | 0.2 | 0.3 | 0.1 | 0.1 | 53.7 |
| **69** | TL | Sävar | 37.8 | 42.3 | 6.8 | 7.3 | 3.0 | 2.6 | 0.1 | 0.0 | 0.0 | 0.1 | 0.0 | 0.0 | 0.0 | 0.0 | 0.0 | 0.0 | 0.0 | 0.0 | 0.0 | 30.5 |
| **72** | TL | Sävar | 39.6 | 40.8 | 3.4 | 1.3 | 8.2 | 5.9 | 0.0 | 0.4 | 0.0 | 0.1 | 0.0 | 0.0 | 0.0 | 0.0 | 0.0 | 0.0 | 0.2 | 0.0 | 0.1 | 26.0 |
| **76** | CN | Sävar | 39.9 | 29.2 | 4.3 | 1.5 | 0.7 | 0.3 | 0.1 | 0.0 | 10.5 | 13.0 | 0.1 | 0.2 | 0.0 | 0.0 | 0.0 | 0.0 | 0.2 | 0.0 | 0.0 | 47.7 |
| **77** | TL | Sävar | 36.5 | 43.7 | 8.3 | 7.7 | 1.4 | 0.8 | 0.1 | 1.3 | 0.0 | 0.0 | 0.0 | 0.0 | 0.0 | 0.0 | 0.0 | 0.0 | 0.1 | 0.0 | 0.0 | 57.1 |
| **78** | TL | Sävar | 45.7 | 40.4 | 6.6 | 3.1 | 1.7 | 0.7 | 0.0 | 0.6 | 0.0 | 0.0 | 0.0 | 0.0 | 0.0 | 0.0 | 0.0 | 0.0 | 0.8 | 0.2 | 0.2 | 20.1 |
| **79** | TL | Sävar | 35.3 | 42.7 | 7.5 | 1.5 | 7.3 | 3.5 | 0.0 | 0.1 | 0.0 | 0.1 | 0.0 | 0.0 | 0.0 | 0.0 | 0.0 | 0.0 | 1.2 | 0.5 | 0.4 | 12.4 |
| **88** | CN | Sävar | 37.6 | 19.3 | 7.2 | 2.3 | 3.2 | 1.0 | 0.1 | 0.5 | 12.0 | 14.7 | 0.4 | 0.3 | 0.2 | 0.7 | 0.1 | 0.1 | 0.3 | 0.0 | 0.0 | 45.2 |
| **97** | CN | Sävar | 34.3 | 20.5 | 10.2 | 3.9 | 6.0 | 2.2 | 0.1 | 0.0 | 8.9 | 12.4 | 0.6 | 0.3 | 0.0 | 0.0 | 0.1 | 0.3 | 0.2 | 0.0 | 0.0 | 30.0 |
| **100** | TL | Sävar | 35.0 | 41.9 | 7.7 | 7.7 | 2.9 | 1.2 | 0.1 | 2.0 | 0.0 | 0.1 | 0.0 | 0.0 | 0.0 | 0.0 | 0.0 | 0.0 | 0.9 | 0.3 | 0.3 | 46.2 |
| **110** | TL | Sävar | 44.9 | 44.4 | 3.4 | 2.4 | 1.9 | 1.0 | 0.1 | 1.6 | 0.0 | 0.0 | 0.0 | 0.0 | 0.0 | 0.0 | 0.0 | 0.0 | 0.2 | 0.0 | 0.0 | 54.9 |
| **115** | AC | Sävar | 35.3 | 37.1 | 2.4 | 1.6 | 1.2 | 0.6 | 0.1 | 1.5 | 0.0 | 0.1 | 0.0 | 0.0 | 0.0 | 0.0 | 0.0 | 0.0 | 18.0 | 1.5 | 0.7 | 60.6 |
